# Kinetic and Chemical Effects of Clays and Other Fillers in the Preparation of Epoxy–Vinyl Ether Composites Using Radical-Induced Cationic Frontal Polymerization

**DOI:** 10.1021/acsami.3c00187

**Published:** 2023-04-07

**Authors:** Brecklyn R. Groce, Emma E. Lane, Daniel P. Gary, Douglas T. Ngo, Dylan T. Ngo, Fahima Shaon, Jorge A. Belgodere, John A. Pojman

**Affiliations:** † Department of Chemistry and the Macromolecular Studies Group, Louisiana State University, Baton Rouge, Louisiana 70803, United States; ‡ Department of Biological and Agricultural Engineering, Louisiana State University, Baton Rouge, Louisiana 70803, United States

**Keywords:** frontal polymerization, cationic polymerization, fillers, clays, minerals, kinetics, epoxies, vinyl ethers

## Abstract

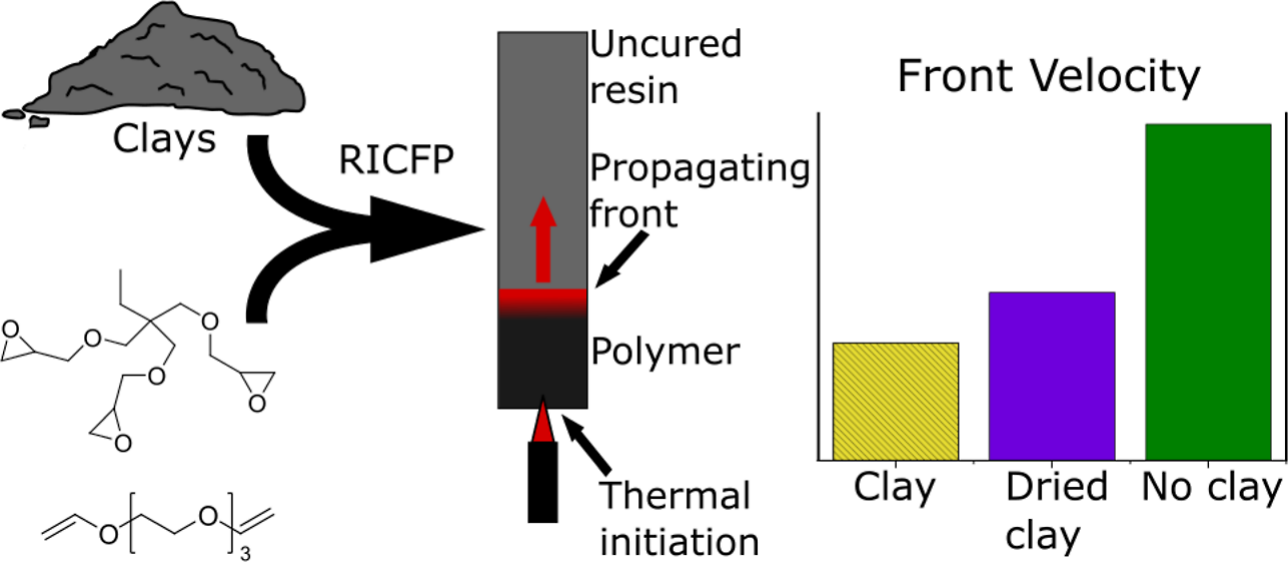

Addition of fillers to formulations can generate composites with improved mechanical properties and lower the overall cost through a reduction of chemicals needed. In this study, fillers were added to resin systems consisting of epoxies and vinyl ethers that frontally polymerized through a radical-induced cationic frontal polymerization (RICFP) mechanism. Different clays, along with inert fumed silica, were added to increase the viscosity and reduce the convection, results of which did not follow many trends present in free-radical frontal polymerization. The clays were found to reduce the front velocity of RICFP systems overall compared to systems with only fumed silica. It is hypothesized that chemical effects and water content produce this reduction when clays are added to the cationic system. Mechanical and thermal properties of composites were studied, along with filler dispersion in the cured material. Drying the clays in an oven increased the front velocity. Comparing thermally insulating wood flour to thermally conducting carbon fibers, we observed that the carbon fibers resulted in an increase in front velocity, while the wood flour reduced the front velocity. Finally, it was shown that acid-treated montmorillonite K10 polymerizes RICFP systems containing vinyl ether even in the absence of an initiator, resulting in a short pot life.

## 1 Introduction

Frontal polymerization (FP) is a method in which, through the coupling of Arrhenius rate kinetics of an exothermic polymerization and heat diffusion, a localized reaction zone will propagate through unreacted resin until it polymerizes the entirety of the starting material.^1^ It was first discovered in Russia in the 1970s^2^ and studied before the FP of methacrylic acid was independently rediscovered by Pojman in 1991 in the US.^3^ FP has since also been used in ring-opening metathesis polymerization^4−9^ and cationic polymerization^10−14^ and developed for different applications, such as composites,^10,15−17^ additive manufacturing,^18−20^ and adhesives.^12,21^

**Scheme 1:**
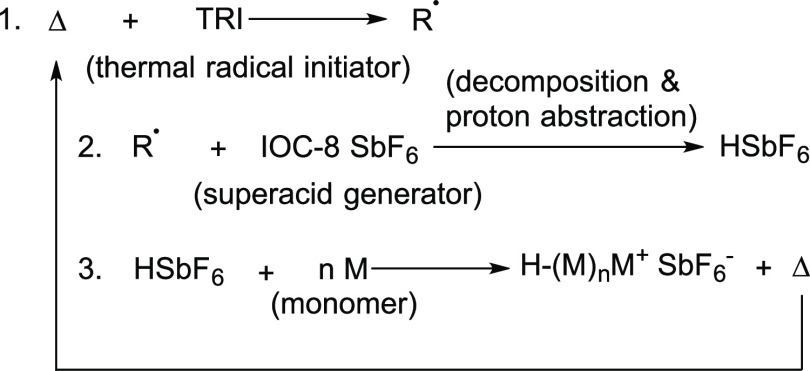
Simplified Mechanism of RICFP with an Iodonium-Based Superacid Generator

RICFP is a newer development in FP, where polymerization proceeds through a cationic mechanism rather than the typical free-radical processes. This process allows for the FP of epoxies without using amine curing agents that give formulations short pot lives.^22,23^ Epoxies are commonly used in composites and adhesives. RICFP was developed by Mariani et al. in 2004,^24^ and almost all focus has been on studying epoxy resins and composite materials.^14,17,25−27^ RICFP proceeds through the mechanism shown in Scheme 1, where it can be seen that it combines cationic polymerization with radical FP through the integration of a thermal radical initiator, typically a peroxide or benzopinacol,^28^ with an acid generating salt, typically an antimonate-based iodonium salt.

A phenomenon that can quench a propagating front is convection. Buoyancy-driven convection is always present in fronts carried out horizontally due to the large gradients of temperature and concentration.^1^ An increase of viscosity can suppress the effects of convection.^1,29−34^ The addition of fumed silica is a simple way to raise the viscosity. Fumed silica has been applied to both free-radically and cationically curing resins to stop convection.^35,36^ The addition of fillers can suppress convection, enhance mechanical properties, modify rheological properties, and reduce cost through a reduction of resin needed.^37,38^

This work will focus mainly on the addition of clay fillers to RICFP resins—Gary et al. have previously studied the effects on acrylate free-radical FP.^36^ They found that kaolin, talc, and calcium carbonate behaved similarly and were chemically inert, while bentonite reduced the front velocity due to both a lower thermal diffusivity and the presence of water, which contributed to heat loss. In addition, it was found that acid-activated clays, such as montmorillonite K10, inhibit free-radical FP.

The minimum requirement for any FP process, irrespective of the mechanism of polymerization, is that heat generation sufficiently exceeds heat loss to both the system and the surroundings.^1^ One source of heat loss is the filler, which acts as a heat sink.^39^ Evaporation of water in swelling clays during FP absorbs heat generated by the system, also contributing to heat loss. Drying clays can eliminate the water present and increase front velocity.^36^ However, removing water from clays opens acidic sites that can act as radical inhibitors or catalyze cationic polymerizations.^40,41^ Two forms of bentonite (Na-bentonite and Ca-bentonite) were found by Gary et al. to affect the front velocity differently through this phenomenon, with the Ca-bentonite likely being more acidic and resulting in a lower front velocity.^36^

For RICFP, there have been a few studies that examined the addition of fillers. Bomze et al. looked at the addition of mica to prove that RICFP is applicable in UV-initiated fronts with filler added.^42^ One of the problems with adding filler to systems is that fillers reduce UV light penetration.^43−45^ Bomze et al. found that up to 15 phr (parts per hundred resin) of mica could be added to BADGE resins with sensitizer added and still support a front. The front velocity did decrease with increasing loadings of mica, explained by the heat absorbed by the filler and the decreased fraction of monomer present. Thermal initiation allowed FP of the epoxy with 15 phr mica and no sensitizer. Klikovits et al. saw reductions in front velocity as SiO_2_ nanopowder was added to BADGE, up to a 10% reduction with 3 phr SiO_2_.^46^ They attributed the inhibition to the low thermal conductivity of the filler, while the insulating properties of the SiO_2_ made initiation of the front quicker. Finally, Tran et al. carried out large studies of fillers with varying thermal properties.^17^ Using BADGE resins with reactive diluent added, they successfully used RICFP to cure polymers with high volume percentages, including 74 vol % glass microspheres. They saw that adding thermally conductive fillers, such as aluminum, short carbon fibers, and graphite, resulted in a smaller front velocity reduction than mica, a mineral which is less conductive. These results are similar to what has been seen for free-radical FP, where milled carbon fibers are shown to increase front velocity as loading increases.^36^ Adding graphite and aluminum to acrylate systems has been shown to reduce the front velocity due to radical scavenging.^36^

With the mechanistic differences between RICFP and free-radical FP, plus the desire to develop practical materials using RICFP, there exists a need to explore more effects of clays on front reactivity with cationic systems. In addition to the differences in thermal characteristics between clays, there are variations in chemistries of clays that could affect propagating cations but not free-radical polymerization. For this study, a monomer system of trimethylolpropane triglycidyl ether and tri(ethylene glycol) divinyl ether was used. Vinyl ethers have been shown to significantly increase front velocity in RICFP systems and were chosen for this purpose.^35^ Using fumed silica as an inert viscosity enhancer allowed controlled amounts of clays to be added to systems. Zoltek-milled carbon fibers and pine wood flour were used to study the effect of differing thermal properties. Drying the clays to increase Lewis acidity and the addition of acid-activated clays were investigated to see any possible chemical effects with the RICFP mechanism.

## 2 Materials and Methods

### 2.1 Materials

**Figure 1:**
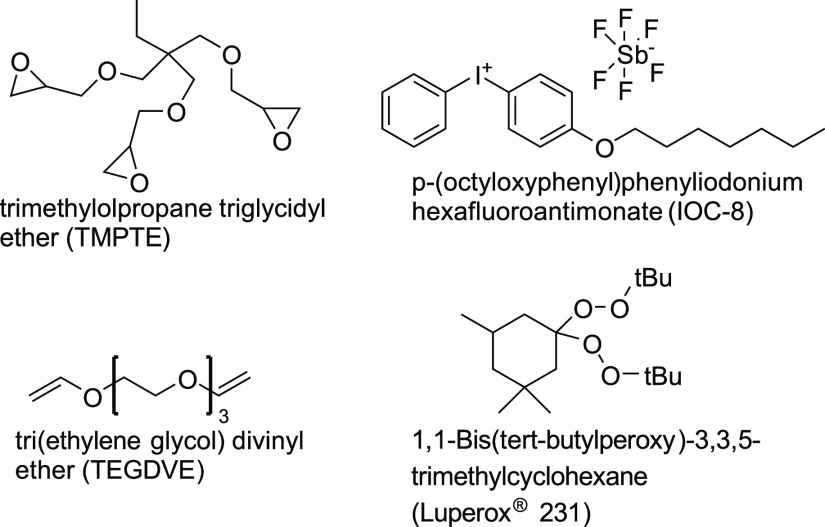
Chemical structures of monomers and initiators used in this study.

Trimethylolpropane triglycidyl ether (TMPTE), tri(ethylene glycol) divinyl ether (TEGDVE), and 1,1-bis(*tert*-butylperoxy)-3,3,5-trimethylcyclohexane (Luperox 231) were purchased from Sigma-Aldrich. (4-(Octyloxy)phenyl) (phenyl)iodonium hexafluorostibate(V) (IOC-8) was purchased from AmBeed, Inc. (Arlington Heights, IL) and Hampford Research, Inc. (Stratford, CT). All chemicals were used as received. The chemical structures of the monomers and initiators used are shown in Figure 1. Talc was purchased from US Composites, Inc. (West Palm Beach, FL). Hectalite 200 (hectorite) and bentonite HPM-20 (Na-bentonite) were purchased from Sheffield Pottery, Inc. (Sheffield, MA). Bentolite L10 (Ca-bentonite) and Fulcat 435 were purchased from BYK Additives & Instruments (Wallingford, CT). Polygloss 90 (kaolin) was purchased from KaMin Performance Materials (Macon, GA), and Hubercarb Q3 (calcium carbonate) was purchased from Huber Materials (Quincy, IL). Zoltek PX35 milled carbon fiber was obtained from Zoltek Companies, Inc. (St. Louis, MO). Nyad G, 10 ES Wollastocoat, and Nyad 1215 were purchased from Imerys Performance Additives (San Jose, CA). Fibertec 520S, Microglass 7204, Microglass 9114, and FRM were provided by Fibertec, Inc. (Bridgewater, MA). Montmorillonite K10 was purchased from Sigma-Aldrich. Wood flour (60 mesh pine flour) was obtained from American Wood Fibers.

### 2.2 Formulation Preparation

Typical formulations consisted of 75 wt % TMPTE and 25 wt % TEGDVE, along with 1 phr (parts per hundred resin) of IOC-8 and 1 phr of Luperox 231, unless otherwise indicated. Parts per hundred resin is a term meaning 1 g of material for every 100 g of resin. IOC-8 was first mixed with TMPTE and sonicated for approximately 15 min to fully dissolve the IOC-8 before addition of TEGDVE and Luperox 231. The solution was then mixed using a vortex mixer for approximately 30 s to ensure homogeneity.

### 2.3 Drying Clays

To dry clays, the clays were placed in an oven containing a desiccant at 200 °C for 3 h before being removed and added to samples once cooled to approximately 30 °C to prevent spontaneous polymerization. The water loss percentage was measured both gravimetrically with a lab balance after the 3 h heating period and using a TA Instruments TGA 550 thermogravimetric analyzer and a Hi-Res heating method. Detailed instrument procedures are available in the Supporting Information.

### 2.4 FP of Formulations

To prepare samples for FP, aliquots were taken from a stock formulation solution, and typically 5 wt % fumed silica [Aerosil 200, Evonik Industries (Parsippany, NJ)] and 30 wt % of the other filler were added to form a moldable putty when hand mixed. Other separate filler loadings were hand mixed with the appropriate amount of filler added as described. Where specified, some formulations were mixed using a FlackTek DAC 515-200 SE speed mixer. The putty was loaded into a wooden mold (13.5 cm × 2 cm × 0.6 cm) lined with wax paper for easy removal of the polymer. A type K thermocouple connected to a handheld thermometer device (Benetech GM1312, purchased from Amazon) was then placed 2 cm into the length of the sample and approximately halfway into the sample depth to record the front temperature. A soldering iron heated to approximately 200 °C (confirmed with an infrared thermometer) was used to initiate the fronts by making brief contact with the sample. The fronts were tracked using a video camera placed directly above the sample. Front velocity was calculated from the slope of front position versus time. The front velocity after expansion, shown in the manuscript as “exp. corrected front velocity”, was calculated by first taking the initial length of the resin in the mold and dividing by the final length of the cured material. The front velocity from the slope of front position versus time was then multiplied by this factor to result in the “exp. corrected front velocity.” The mold was cooled to room temperature prior to subsequent experiments. Triplicate experiments from the same stock were performed for each formulation.

### 2.5 Characterization Methods

Uncured sample viscosity was determined using a TA Discovery HR-2 rheometer. Initially, the plate height was set to 550 μm; excess sample was removed; and the plate height was reduced to 500 μm to ensure total probe coverage. The viscosity (Pa·s) measurements were performed at low (1 Hz) and high (10 Hz) shear rates. The first step was at 1 Hz for 60 s, followed by an increase to 10 Hz for 60 s and returning to 1 Hz for 60 s. Recovery percent was calculated by comparing the viscosities of the first (1 Hz) and last (1 Hz) steps for all formulations. Three replicates were evaluated for each formulation, and the data are presented as the mean ± SD.

Samples of the cured composites for scanning electron microscopy (SEM) and energy dispersive X-ray spectroscopy (EDS) to determine filler distribution were analyzed with a Thermo Scientific Helios G4 PFIB CXe electron microscope. A TA Instruments DSC Q100 differential scanning calorimeter (DSC) was used to determine the glass transition temperature of the cured composite, using a scan rate of 10 °C min^–1^ from −40 to 150 °C and 3 scan cycles. A sample size of approximately 5.5–9.5 mg was used.

Flexural testing was performed on an Instron 5969 universal testing machine. Samples for testing were cut from sheets perpendicular to the direction of front propagation, with sizes according to ASTM D790. Crosshead speed was calculated based on the ASTM standard and the thickness and width of each sample. The support span length was set from 78 to 85 mm based on the sample length.

## 3 Results and Discussion

### 3.1 Comparison of Clays

**Figure 2:**
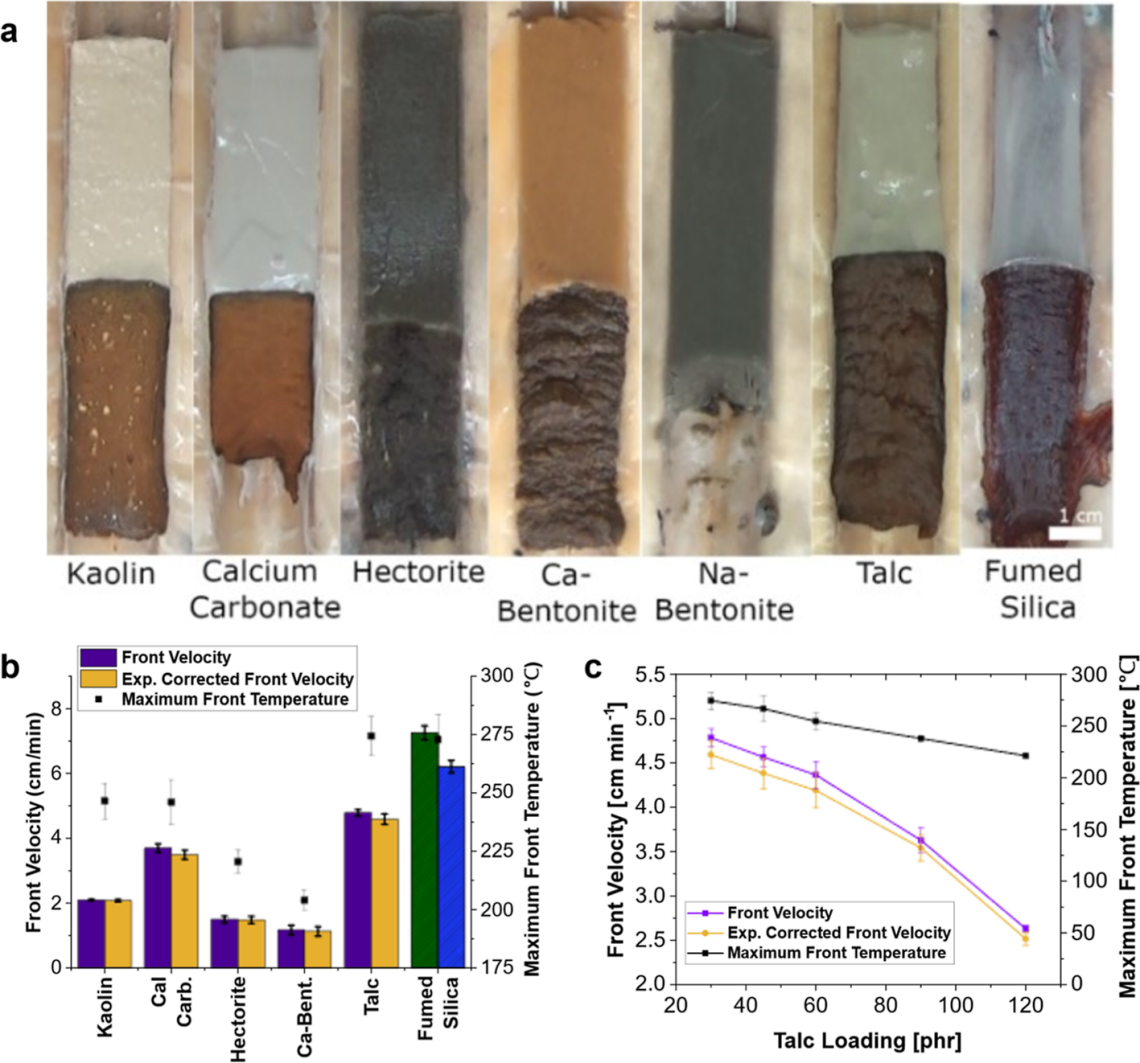
Front kinetics of clay-filled composites using a resin system of 25 wt % TEGDVE, 75 wt % TMPTE, 1 phr IOC-8, and 1 phr Luperox 231. 30 phr of each clay and 5 phr of fumed silica were added to each sample except the “fumed silica” control, shown in a different color and texture, which contained only 5 phr fumed silica. “exp. corrected front velocity” is velocity corrected for sample expansion during front propagation. (a) Resin mixtures with clay fillers added and placed in molds, with an actively propagating front, except in the case of Na-bentonite, which would not support a front. The actively propagating front is seen to have a darker color than the uncured resin. (b) Comparison of front velocities and maximum front temperatures for four clays and calcium carbonate tested with RICFP. (c) Front velocity, expansion corrected front velocity, and maximum front temperature versus talc loading.

Clays were first tested for their effects on front kinetics in RICFP systems. Talc, hectorite, Na-bentonite, Ca-bentonite, kaolin, and calcium carbonate were added in 30 phr amounts to formulations containing the resin system along with 5 phr fumed silica as an additive to increase viscosity and suppress convection. Formulations with clays added, shown in Figure 2a, are placed in molds with propagating fronts in each sample. It was found that all clay fillers tested supported a front except Na-bentonite. Compared to a control containing only 5 phr fumed silica, all clays reduced the front velocity of the system shown in Figure 2b. Calcium carbonate, while not a clay, was used for comparison, and it also reduced the front velocity. These reductions were partially due to heat absorbed by the filler.

The clays all showed differing effects on front velocity. Ca-bentonite was found to reduce the front velocity and front temperature the most, while talc resulted in the least deviation from the control sample for velocity and temperature. Examining talc and kaolin shows a nearly 230% difference in front velocity, while the thermal diffusivities, heat capacities, and thermal conductivities of the two clays are similar, as shown in Table 1. The thermal properties for hectorite are not available; though, we propose that they are similar to the thermal properties of bentonite, as both clays are smectites. Likewise, calcium carbonate is known to have a thermal diffusivity an order of magnitude higher than talc or kaolin, yet the front velocity found lies within the values observed for kaolin and talc. It is likely that the differences in front velocity and temperature among the clays are due to chemical effects, such as interaction of the clays with propagating cations or the superacid, or water content rather than thermal properties. Some clays, such as kaolinites, carry a net negative charge that could be a source of inhibition for RICFP.^47,48^ It has been reported that cationic dyes can adsorb to kaolinites, which supports inhibitory interactions with superacid cations.^49^ The results shown in this work are contrary to previous studies of clays added to free-radical FP systems, where calcium carbonate, kaolin, and talc all behaved similarly. Calcium carbonate was initially hypothesized to not support FP due to its basic character. This was proven otherwise and attributed to the calcium carbonate being mixed into the system as a suspension and not dissolved. Therefore, the basic character of the filler was unable to quench the cationic polymerization. It could still inhibit cationic FP, as it has a lower front velocity than talc while having a higher thermal diffusivity. Some clay fillers have also been shown to act as radical scavengers. The mechanism for RICFP dictates a radical-producing step, which could be inhibited by potential radical scavenging by the clay, resulting in a reduction in front velocity.

**Table 1:** Physical and Thermal Properties of Fillers Used in This Study

	density (g cm^–3^)	heat capacity (J g^–1^ K^–1^)	conductivity (W cm^–1^ K^–1^)	thermal diffusivity (cm^2^ s^–1^)
kaolin^a^^,^^36^	2.6	0.92	2.0 × 10^–2^	8.2 × 10^–3^
calcium carbonate^a^^,^^36^	2.7	0.87	2.5 × 10^–2^	1.1 × 10^–2^
talc^a^^,^^36^	2.8	0.87	2.1 × 10^–2^	8.6 × 10^–3^
bentonite^a^^,^^36^	2.6	1.30	1.0 × 10^–2^ 1.3 × 10^–2^	3.0 × 10^–3^–3.9 × 10^–3^
wood flour^50^	0.4–1.35	1.75	0.25 × 10^–2^	3.6 × 10^–3^–1.1 × 10^–3^
milled carbon fiber^a^^,^^36^	1.8	0.60	6.4 × 10^–2^	5.9 × 10^–2^
fumed silica^a^^,^^36^	2.2	0.79	1.5 × 10^–4^	8.6 × 10^–5^

a Provided by the manufacturer.

The effects of adding increasing amounts of talc and hectorite were investigated. It was found that for both talc and hectorite, adding more clay filler resulted in decreasing front velocity and front temperature, as shown in Figure 2c for talc. A loading of up to 120 phr of talc was tested, and the front was still viable. However, at only 32 phr hectorite, the front would quench after a few centimeters of propagation. Overall, adding hectorite resulted in much lower front velocities than adding talc. The differences in maximum possible loadings and lower front velocities with hectorite are attributed again to water content and chemical effects. Decreasing the front temperature and front velocity points to both the water content and the clays acting as heat sinks, leading to decreased reactivity. The trend of decreasing front velocity with increasing filler loadings has been previously reported by Tran et al. for RICFP systems and Scognamillo et al. for a BF_3_-amine-cured epoxy system.^17,22^

**Figure 3:**
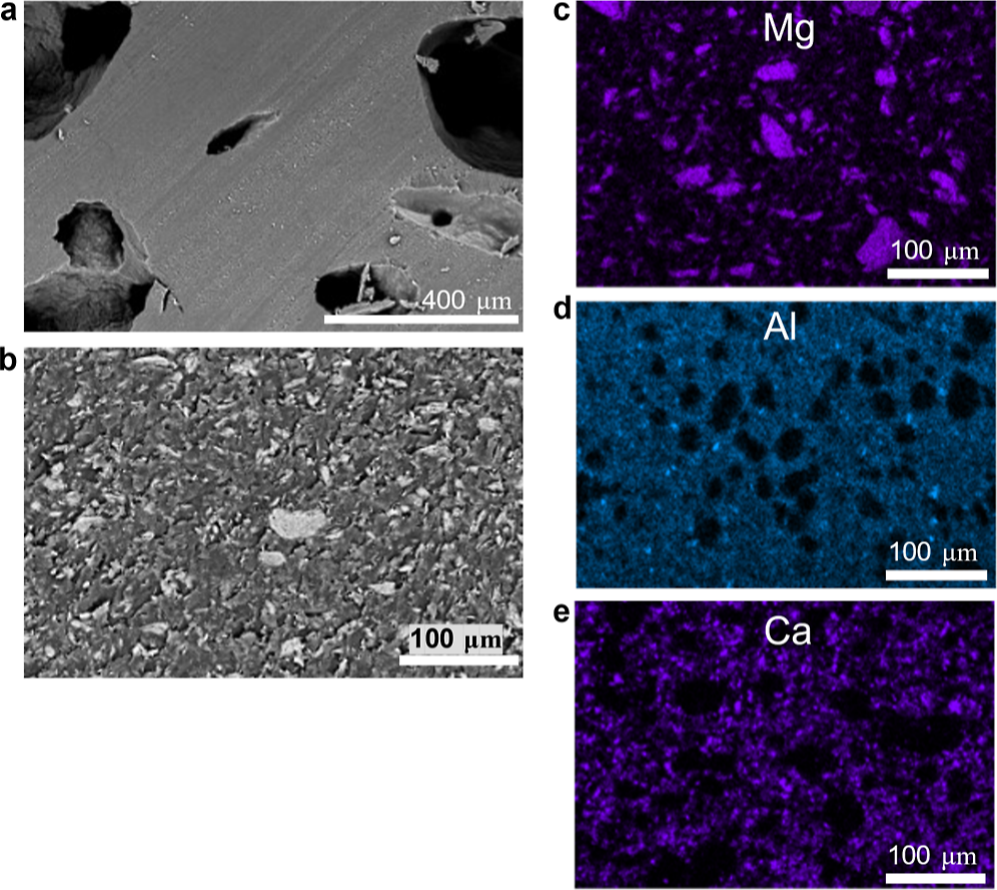
Microscopy imaging of filled composites. All contain the same resin formulation of 25 wt % TEGDVE, 75 wt % TMPTE, 1 phr IOC-8, and 1 phr Luperox 231 along with 5 phr fumed silica plus indicated filler, except in the case of (a). (a) SEM image of 10 phr fumed silica with visible voids. (b) Composite with 30 phr talc. (c) EDS map of Mg present in a 30 phr talc composite. (d) EDS map of Al present in a 30 phr kaolin composite. (e) EDS map of Ca present in a 30 phr calcium carbonate composite.

SEM–EDS analysis of the cured resins by examining characteristic elements with EDS maps showed that the dispersion of the fillers varied with the selected filler. Ca-bentonite, hectorite, and talc had some larger particles remaining in addition to portions of the unfilled polymer. Calcium carbonate was homogeneously distributed in the system, while kaolin appeared to be distributed only in specific sections of the system. The dispersion using a speed mixer versus hand mixing was also analyzed. In the case of 30 phr talc, large particles remained, but the filler appeared more homogeneously distributed. For all other fillers, there were insignificant differences in the distribution when speed was mixed. A significant presence of chlorine was also observed with EDS in the polymer region, which we attribute to impurities present during the production of the epoxy monomer. The EDS maps of talc, kaolin, and calcium carbonate-containing composites are shown in Figure 3. Additional figures are available in the Supporting Information.

#### 3.1.1 Effects of Viscosity on the Front Velocity

**Figure 4:**
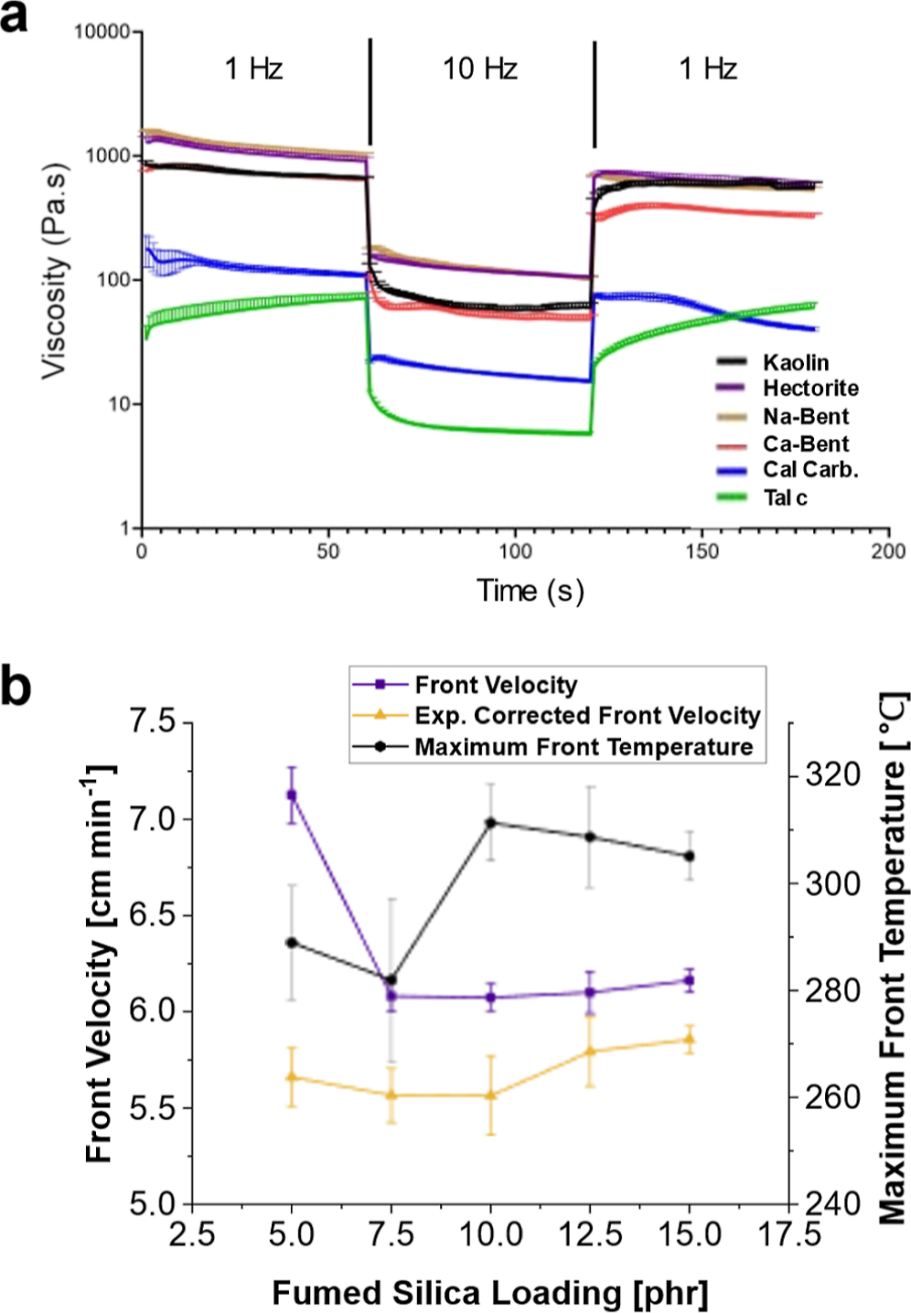
Front kinetics dependent on viscosity. (a) Viscosity profile of formulations containing the resin system of 25 wt % TEGDVE, 75 wt % TMPTE, 1 phr IOC-8, and 1 phr Luperox 231, with 5 phr fumed silica and 30 phr of specified clay. The first shear rate was 1 Hz, followed by 10 and 1 Hz, with each shear rate held for 60 s at 25 °C. “Ca bent” is calcium bentonite, “Na bent” is sodium bentonite, and “cal carb” is calcium carbonate. (b) Front velocity, front velocity corrected for expansion, and front temperature as a function of fumed silica loading. “exp. corrected front velocity” is velocity corrected for sample expansion during front propagation.

To discern potential impacts of viscosity on the front velocity, the amount of fumed silica was varied from 5 to 15 phr in the absence of other fillers, which produces a significant increase in viscosity, as shown in Figure S6. Since clays have different densities and properties, they can affect the overall viscosity of formulations differently. The viscosity profile of each clay formulation was evaluated and shown in Figure 4a. The differences in viscosity based upon which clays are added and hand-mixed into the formulation can be clearly seen. All the formulations exhibited shear-thinning thixotropic behavior with a recovery of viscosity after application of high shear. The recovery of viscosity was calculated and shown in Supporting Information. Recovery appears to decrease as the talc loading is increased. Since samples for characterization were made using a speed mixer rather than hand mixing, comparisons in the viscosity profile were also examined. It was found that speed-mixed samples containing kaolin and talc have both a lower viscosity and more deviation in their viscosities. Notably, speed-mixed samples with 30 phr talc and 5 phr fumed silica were difficult to work with due to their much lower viscosity. They also exhibited a unique phenomenon that appeared to be a phase separation of high and low viscosity portions after speed mixing. However, comparing front velocities in Figure S10 of hand and speed mixed samples showed that the values overall vary slightly, but the trends remain the same.

As shown in Figure 4b, the front velocity decreased from 7.3 cm min^–1^ with 5 phr fumed silica to 6.1 cm min^–1^ with 7.5 phr fumed silica. The front velocity then remained constant with increasing additions. The higher front velocity with less viscous material is expected with FP, where previous studies have shown that convection could affect front propagation.^1,29^ Correcting for the expansion of the sample as the front propagated gave a weak dependency of front velocity across all fumed silica loadings tested, which was initially expected. The front temperature appeared nearly constant with fumed silica loadings of 10 to 15 phr, and the lower front temperature at 5 and 7 phr loadings could be indicative of convection giving rise to heat loss. We tested a system with 30 phr calcium carbonate while varying the fumed silica loading from 5 to 7 phr. The qualitative increase in viscosity resulted in a statistically insignificant difference in front velocity. 5 phr calcium carbonate gave an expansion-corrected front velocity of 3.49 ± 0.11 cm min^–1^, while 7 phr resulted in 3.43 ± 0.09 cm min^–1^. Calcium carbonate was chosen because it is not a clay, so there are no chemical effects regarding swelling or acidic sites. With these studies, it was concluded that, above a threshold for eliminating buoyancy-driven convection, the viscosity has no significant effect on the front velocity. Therefore, it is concluded that the trends observed with increasing clay loadings are not related to viscosity.

### 3.2 Effects of Drying Clays

**Figure 5:**
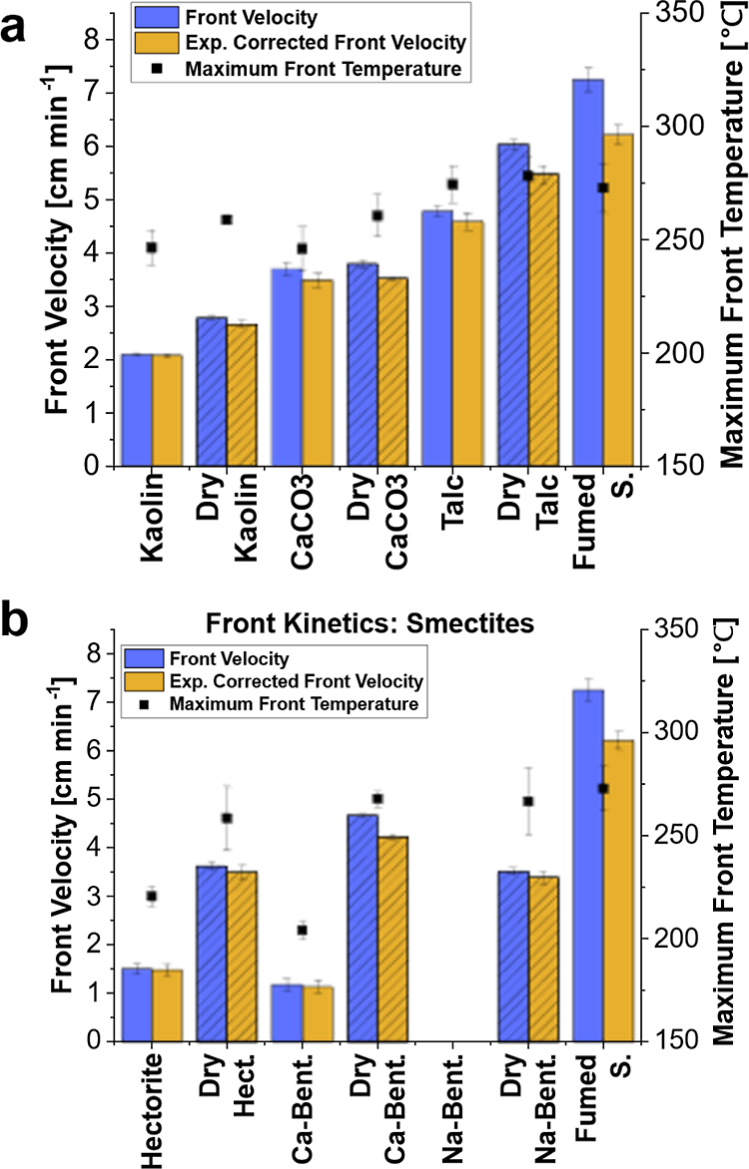
Comparison of dried and as-received clays tested for front velocity and front temperature. Samples contained 30 phr clay and 5 phr fumed silica added to a resin system of 25 wt % TEGDVE, 75 wt % TMPTE, 1 phr IOC-8, and 1 phr Luperox 231. “fumed S.” contained only 5 phr fumed silica added with no clay. Dried fillers are shown in a filled pattern. “exp. corrected front velocity” is velocity corrected for sample expansion during front propagation. (a) Front kinetics comparisons with dry and as-received clays and calcium carbonate. (b) Front kinetics comparisons with dry and as-received smectite clays. “Hect.” is hectorite, and “Bent.” is bentonite.

Drying the clays had substantial effects on the front velocity and temperature and produced an overall increase in front velocity and temperature in all cases, as shown in Figure 5a,b for the smectite clays. The differences are attributed to the water content of the clays, which can be seen as a percentage weight change measured gravimetrically and with a thermogravimetric analyzer (TGA), compared with the manufacturer reported moisture content in Table 2.

**Table 2:** Water Loss after Drying and Measured through Gravimetric and Thermogravimetric Analysis

	wt % water loss	wt % water loss (TGA)	reported moisture content (wt %)^a^
kaolin	1.3	0.74	1.0
calcium carbonate	0.15	0.32	0.2
hectorite	8.0	7.1	<12
Ca-bentonite	13.9	11.4	<8.0
Na-bentonite	8.4	7.2	<12
talc	0.25	0.61	not provided

a From manufacturer technical data sheet.

The TGA curves for each clay are available in Supporting Information, as shown in Figures S11–S16. The water loss observed with TGA was an average of two runs and analyzed over a range of 25 to 200 °C to best mimic the oven drying process. Both talc and calcium carbonate saw a sudden weight percent increase of approximately 0.4 and 0.7% from 25 to 50 °C, respectively, across two separate runs. Thus, the water loss calculated from the TGA curve for talc and calcium carbonate used the weight percentage from 50 to 200 °C rather than the initial weight percentage at room temperature. The cause of this sudden increase is unknown to the authors; the thermal analysis of clay decomposition is beyond the scope of this article.

Hectorite, sodium bentonite, and calcium bentonite exhibited the highest changes upon drying, which coincides with their much higher water content than the other clays tested.^36^ Notably, Na-bentonite would not support a front until it dried.

Drying could result in a few possibilities that would both increase the front velocity. First, a higher water content could mean more heat loss in the system due to the vaporization of water. Analyzing the maximum front temperatures reveals that for every clay tested, drying resulted in a temperature almost identical to that of the fumed silica control, while the nondried clays had much lower front temperatures. This supports the hypothesis that heat loss from vaporization of water is mitigated by drying the clays. Second, drying clays has been associated with freeing up acidic sites.^40,41,51^ It is possible that this is catalyzing the RICFP rather than acting as an inhibitor for the process, as seen with previous works based on radical FP. Finally, water could inhibit the cationic propagation reaction. The reduction in front velocity with calcium carbonate compared to the fumed silica control can be attributed to potential chemical interaction with propagating cations or the increase in heat capacity of the formulation and heat losses to the calcium carbonate, as shown by the reduction in maximum front temperature compared to the fumed silica control. The constant front velocity, independent of the drying of calcium carbonate, is due to a low amount of bound water.

With the clay fillers, the similar front temperatures after drying compared to the control containing no clay imply that the reduction in front velocity is mostly due to chemical effects or the vaporization of water during propagation rather than heat loss to the clay itself. If the heat loss was due to the thermal properties (such as heat capacity) of the fillers themselves rather than water loss, one would expect the front temperature to be significantly lower than the fumed silica, irrespective of drying.

### 3.3 Material Characterization of Clay-Filled Composites

To study the effects of the fillers on final material properties, testing with DSC to determine glass transition temperature, *T*_g_, and flexural testing were undertaken, with results in Table 3. Composites containing clays of each type, such as kaolinite, calcium carbonate, talc, and smectites, were chosen for characterization. An increase in talc loading was examined to show the effects on material properties with increasing filler.

**Table 3:** Mechanical and Thermal Properties of Composites

	flexural strength (MPa)	flexural modulus (MPa)	flexural strain at break (mm mm^–1^)	glass transition temperature (°C)
fumed silica	15.23	1.20	0.110	–3.95
30 phr talc	8.18	0.55	0.085	–6.16
60 phr talc	31.20	1.78	0.071	–6.81
120 phr talc	156.3	4.26	0.035	2.75
kaolin	43.89	2.73	0.069	–13.05
calcium carbonate	17.14	0.90	0.062	–12.16
hectorite	N/A	N/A	N/A	–15.40

**Figure 6:**
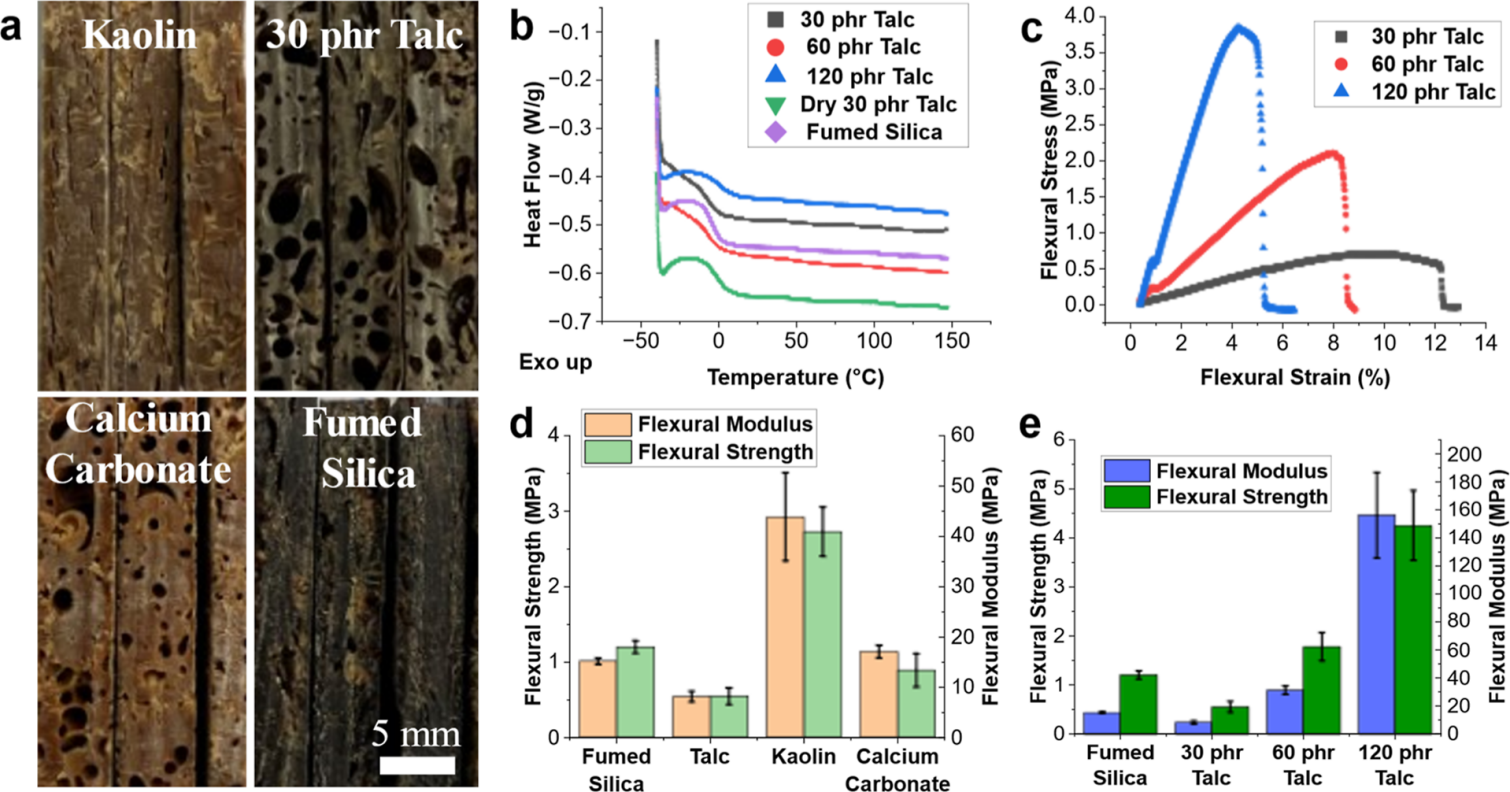
Thermal and mechanical characterization of prepared composites. All composites contain 5 phr fumed silica in addition to filler, except those labeled “fumed silica.” (a) Cross section of composite sheets filled with specified filler with noticeable voids in calcium carbonate and talc. (b) DSC curves from −40 to 150 °C of various talc loadings from 30 to 120 phr, 30 phr dried talc, and 15 phr fumed silica. (c) Flexural stress–strain curve for composites with 30 to 120 phr talc. (d) Flexural modulus and strength for talc, kaolin, calcium carbonate, and fumed silica only sample. (e) Flexural modulus and strength for composites with only fumed silica and composites with 30 to 120 phr talc.

It was found that the *T*_g_ for all studied clay composites was subzero, except in the case of 120 phr talc with 5 phr fumed silica, which still had a *T*_g_ significantly below room temperature, as shown in Table 3. There was a notable decrease in *T*_g_ after the addition of clays compared to a system with only fumed silica. The low *T*_g_ values are not unexpected, as the materials are rubbery and flexible in ambient conditions. The water content of the clay could be playing a role in the low *T*_g_, with hectorite having the lowest measured *T*_g_, and talc, the highest. Additionally, a composite with 30 phr dried talc gave a difference of 4 °C compared to using as-received talc. Calcium carbonate, with the lowest water content of the tested fillers, does not follow this trend of water content, however. There was an increase in *T*_g_ from 30 phr talc to 120 phr talc, likely due to the decreased chain mobility in the system with a high filler content. In most DSC curves shown in Figure 6b, there appears to be a significant exothermic hook from −40 to −20 °C, which was examined and found to be due to improper matching of the reference pan weight with the sample pan weight and not related to the thermal properties of the sample.

Flexural testing was chosen for mechanical testing due to the inherent homogeneities in these systems, with the decomposition of the radical initiator generating voids in addition to the flexible nature of the composites. Hectorite was unable to be studied as there were issues with front propagation in thin sheets for sample preparation. It was found that each clay affects the mechanical properties differently, with the highest flexural strength observed with kaolin and the lowest with 30 phr talc. Only in the case of the 30 phr talc addition was the flexural strength lower than that of fumed silica alone seen in Figure 6e. This is due to the presence of many voids in this composite compared to only fumed silica, which aid in the flexibility of the material but negatively affect its strength. The presence of voids can be seen in Figure 6a. The flexural modulus results in Figure 6d show that the addition of 30 phr talc and calcium carbonate reduced the stiffness of the composite, which could be due to more voids present in these composites compared to the others and fumed silica alone. An increase in talc from 30 to 120 phr showed significant increases in both the flexural strength and flexural modulus. This was coupled with a decreasing strain at break when increasing the talc loading. The flexural stress–strain curve for increasing talc loading is shown in Figure 6c.

### 3.4 Addition of Fillers with Different Thermal Properties

**Figure 7:**
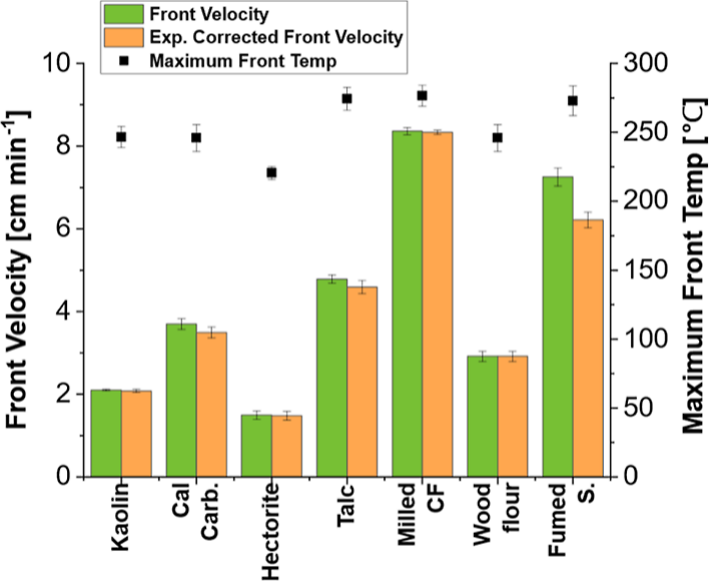
Comparison of fillers with differing thermal properties. Samples contained 30 phr shown filler and 5 phr fumed silica added to a resin system of 25 wt % TEGDVE, 75 wt % TMPTE, 1 phr IOC-8, and 1 phr Luperox 231. “fumed silica” contained only 5 phr fumed silica added with no other fillers. Front velocities and temperatures are shown. “milled CF” is milled carbon fiber and “cal carb.” is calcium carbonate. “exp. corrected front velocity” is velocity corrected for sample expansion during front propagation.

Wood flour and milled carbon fibers were tested to determine the effects of thermal properties on the reactivity of the systems. It was found that wood flour, an insulating filler with a higher heat capacity than the clays and milled carbon fibers tested, lowered the front velocity by 100% and the front temperature by 26 °C, while the addition of carbon fibers gave an increase in front velocity, as shown in Figure 7, with a comparison to the as-received clay fillers studied. It has been reported for FP that adding carbon fibers and other conductive elements will increase front velocities.^9,16,36^ As seen in Table 1, carbon fibers have a thermal diffusivity an order of magnitude higher than wood flour and a higher thermal conductivity. The thermal properties of the carbon fibers aid in the heat diffusion needed to sustain a front, resulting in higher front velocity, while maintaining a constant front temperature compared to the fumed silica control. Wood flour’s low thermal diffusivity reduces the front velocity and, subsequently, the front temperature because the slower front allows for more heat loss.

### 3.5 Effect of Filler Dimensions and Sizings

A small study to discern the effects of the aspect ratio, the ratio of length to diameter, and the surface area of fillers was attempted. Seven fillers were studied, with their properties shown in Table S1: Nyad G, 10 ES Wollastocoat, Nyad 1215, and Fibertec 520S are all produced from wollastonite, a mineral that occurs as needle-like structures. Microglass 7204 and Microglass 9114 are milled e-glass filaments, and FRM is made of mineral wool fibers. The front velocity and temperature results from this study of surface area and dimensions are shown in Figures S18 and S19. No obvious trend in front velocity or temperature was found with the increasing aspect ratio of these fillers. There are two issues affecting these results. The surface area of every filler is not known, but for the known fillers, surface area may play a greater role than the aspect ratio. When arranging the results of fillers with known surface area, it is seen that front velocity decreases as surface area increases. This could be due to the chemistry of the fillers’ surfaces inhibiting the cationic polymerization or differences in the thermal properties of the filamentous fillers. Many of these filamentous fillers are surface treated, which could be affecting propagation. The chemical composition of many surface treatments or sizings is not provided by the manufacturer, and the fillers with known surface areas that were studied have different sizings.

## 4 Effects of Initiator Concentrations and Vinyl Ether Content

**Figure 8:**
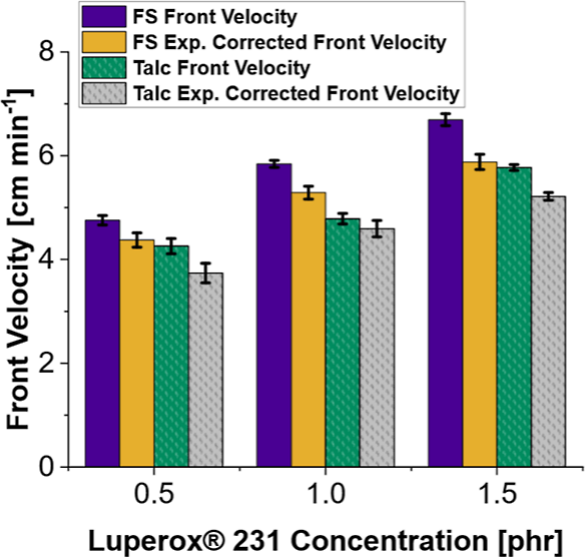
Effect of increasing Luperox 231 concentration on front velocity for formulations containing 25 wt % TEGDVE, 75 wt % TMPTE, and 1 phr IOC-8. Samples contained 10 phr fumed silica (FS) or 5 phr fumed silica with 30 phr talc. “exp. corrected front velocity” is velocity-corrected for sample expansion during front propagation.

Samples with talc, Ca-bentonite, and kaolin were tested with differing amounts of TEGDVE and initiators. Given the ability for clays to show radical-scavenging behavior, it was suspected that increasing initiator concentrations in the presence of clay would result in smaller increases in front velocity than samples with no clay. To study the increases in front velocity, we determined the slope of the linear trendline for front velocity versus Luperox 231 concentration for samples with and without clays. The compiled data from this study are shown in Table 4. From a graph of front velocity for fumed silica and talc with fumed silica samples with increasing Luperox 231 concentration from 0.5 to 1.5 phr shown in Figure 8, it was found that the slope of the linear trendline for the front velocity in fumed silica only samples was 1.94 (cm min^–1^ phr^–1^), and the slope of expansion corrected front velocity was 1.51 (cm min^–1^ phr^–1^). For talc samples, the front velocity and expansion corrected front velocities were 1.52 and 1.47 (cm min^–1^ phr^–1^), respectively. The similarity in front velocity after expansion correction implies that radical scavenging is not the primary source of propagation inhibition with talc.

**Table 4:** Slope of Trendlines of Front Velocity and Exp. Corrected Front Velocity versus Increasing Luperox 231 Concentrations for Different Clays

	front velocity vs Luperox 231 concentration (cm min^–1^ phr^–1^)	exp. corrected front velocity vs Luperox 231 concentration (cm min^–1^ phr^–1^)
fumed silica^a^	1.94	1.51
fumed silica^b^	2.29	2.51
Ca-bentonite^b^	1.46	1.27
talc^a^	1.52	1.47
kaolin^a^	0.81	0.80

a Luperox 231 concentration increased from 0.5 to 1.5 phr.

b Luperox 231 concentration increased from 1.0 to 2.0 phr.

In the case of Ca-bentonite and kaolin, the slope of the trendline of increasing front velocity versus Luperox 231 for these clays is nearly half that of samples with only fumed silica. The graphs of front velocity versus radical initiator concentration are shown in Figures S20 and S21 for Ca-bentonite and kaolin. Ca-bentonite has been previously shown to affect free-radical FP through potential radical inhibition and low thermal diffusivity.^36^ A front after adding Ca-bentonite was not supported with 1.0 phr IOC-8 and 0.5 phr Luperox 231; therefore, an increasing Luperox 231 range of 1.0 to 2.0 phr was chosen. The lower slope of Ca-bentonite in this study indicates either radical inhibition or low thermal diffusivity.

Kaolin and talc have previously been shown to be inert in free-radical FP systems.^36^ The front velocity for kaolin is nearly half that of talc in RICFP systems which, with their similar thermal properties, shows potential chemical inhibition. Although previously shown to be inert in free-radical FP, the slope of the trendline of increasing radical initiator concentration was much less after adding kaolin. This could indicate that the mechanism of inhibition with kaolin is through interaction with free radicals, contrary to previous work which contrasted kaolin, talc, calcium carbonate, and bentonite.

With 0–100% TEGDVE/TMPTE, an overall increase in front velocity for samples containing talc was observed with the increasing TEGDVE content, which is an expected trend.^35^ At 25% TEGDVE, the front velocity did decrease, which was also observed in previous reports.^35^ For samples with hectorite, a front was not supported with 100% TMPTE and no TEGDVE, though a localized reaction occurred upon application of heat. This could be attributed to the low reactivity of TMPTE relative to vinyl ether and the low thermal diffusivity of hectorite, in addition to the high water content of hectorite contributing to heat loss and inhibition of the cationic mechanism. Given that talc would support a front with only TMPTE and the initiators, we suspect this to be a result of the unique chemistry of the hectorite.

## 5 Spontaneous Polymerization Induced by Montmorillonite K10

Montmorillonite K10 (MMT K10), an acid-treated bentonite clay, was able to initiate polymerization in the absence of any applied external energy, resulting in many localized fronts in the material. The rapid initiation, with a decreasing onset time as MMT K10 loading is increased, is contrary to previous reports of filler addition in free-radical FP.^36^ This is attributed to the acidic character of the clay, as it is well known that vinyl ethers and other monomers will polymerize upon addition of acid;^52^ acid-treated montmorillonites have also been used as catalysts for cationic polymerizations and organic reactions.^53−55^ It was found that a minimum of 10 phr MMT K10 was needed to initiate polymerization. With free-radical FP, MMT K10 and other acid-treated clays were shown to inhibit the FP process or even quench it at high loadings,^36^ while in this case for cationic monomers, the acidic character is resulting in the initiation of polymerization.

**Figure 9:**
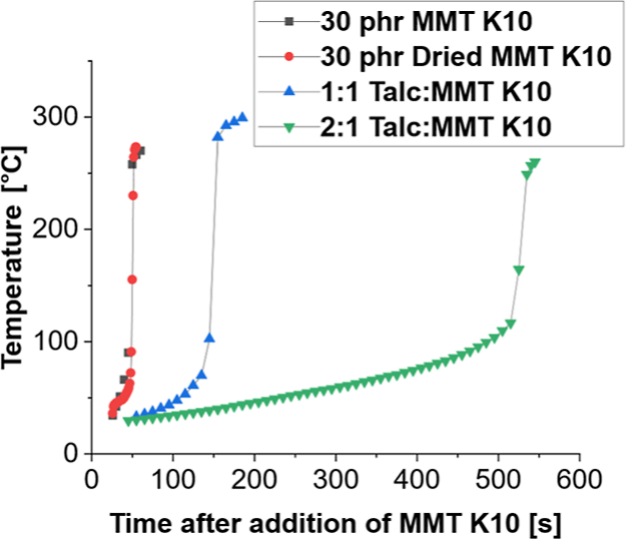
Effects of talc and MMT K10 addition along with drying on the pot life of 25 wt % TEGDVE, 75 wt % TMPTE with 1 phr IOC-8, and 1 phr Luperox 231 formulations.

Increasing the ratio of talc to MMT K10 resulted in a prolonged initiation time. Drying the clay with the above mentioned procedure did not affect the starting time. A similar result was achieved by Gary et al., who saw that the front velocity of acrylates was unaffected by the drying of Fulcat 435, another acid-activated clay.^36^ The addition of fumed silica also did not significantly affect the pot life. The initiation time based on drying and talc addition is shown in Figure 9. Observing the front velocity of high loadings of MMT K10 was unachievable, given the short pot life until spontaneous polymerization. Likewise, with these formulations, it was impossible to predict the starting location of a front, making the propagation difficult to track.

To confirm that this phenomenon is due to acid treatment and not another property of the clay, tests on identical samples were carried out, but Fulcat 435, another acid-treated clay, was added. The addition of this clay also resulted in spontaneous polymerization. Additions of other untreated clays did not show spontaneous polymerization.

Formulations containing only TMPTE and MMT K10 did not exhibit spontaneous polymerization within similar timespans. This indicates that interactions between the vinyl ether and MMT K10 are causing the polymerization. The nucleophilic double bonds of vinyl ethers are known to be easily protonated.^56^ Like the concept of kick-starting in photopolymerized vinyl ether–epoxy blends,^57,58^ the vinyl ether reactivity drives the polymerization to occur upon protonation via MMT K10.

## 6 Conclusions

We investigated the effects of adding fillers, with a focus on clay minerals, to a resin system of epoxy and vinyl ether, which undergoes RICFP through a combination of an iodonium salt and a peroxide-based free-radical thermal initiator to generate composites. Of the clay minerals tested, talc, kaolin, hectorite, calcium carbonate, and Ca-bentonite all supported fronts with fumed silica added as a viscosifier, while Na-bentonite did not support a front. These clays all decreased the front velocity differently, contrary to reports for free-radical FP. It was shown by increasing the radical initiator concentration that Ca-bentonite and kaolin may be suppressing RICFP through scavenging of radicals. Drying all clays increased the front velocity due to the liberation of water that would otherwise contribute to heat loss and inhibit cationic polymerizations. Increasing filler loading was found to increase the flexural strength, stiffness, and glass transition temperature of the composites. Montmorillonite K10, an acid-treated bentonite, was shown to cause spontaneous polymerization, making it unsuitable for practical applications.

Knowledge of the effects of different fillers on FP is important for the development of composite materials using epoxy or vinyl ether resins. Fillers can have different effects on RICFP rather than free-radical FP. We have shown that choosing the correct filler entails a balance of thermal properties, chemical effects, and water content. Carbon fibers were shown to have much higher front velocities than wood flour due to the much higher thermal diffusivity of carbon fibers. Filamentous fillers with sizings or acid treatment could create systems with different pot lives and front velocities than systems created with untreated fillers.

## Supporting Information Available

30 phr talc front (MOV)
30 phr calcium carbonate front (MOV)
Front velocity as a function of hectorite loading; EDS maps of Mg in 60 phr talc, Mg in 120 phr talc, Al in 30 phr Ca-bentonite, and Al in 30 phr hectorite; viscosity profiles of increasing fumed silica, increasing talc, and hand versus speed mixed kaolin and talc; percent recovery of increasing talc; comparison of front kinetics of speed and hand mixed; detailed TGA procedure; TGA curves of hectorite, calcium bentonite, sodium bentonite, kaolin, calcium carbonate, and talc; calculation of *T*_g_ with the DSC curve of calcium carbonate; properties of filamentous fillers; front velocity and temperature as a function of the filamentous filler aspect ratio, as a function of filler surface area, as a function of Luperox 231 concentration with calcium bentonite, as a function of Luperox 231 concentration with kaolin, and as a function of vinyl ether; and visual of spontaneous montmorillonite polymerization (PDF)

## Supplementary Material

am3c00187_si_001.mov

am3c00187_si_002.mov
