# Supplementary material for: Kinetic and Chemical Effects of Clays and Other Fillers in the Preparation of Epoxy–Vinyl Ether Composites Using Radical-Induced Cationic Frontal Polymerization
Source: ACS Appl Mater Interfaces. 2023 Apr 7;15(15):19403–13. doi: 10.1021/acsami.3c00187 (PMC10119861; doi:10.1021/acsami.3c00187)
Supplement: Supplementary file 3 — am3c00187_si_003.pdf [file am3c00187_si_003.pdf]

Supporting Information for

Kinetics and Chemical Effects of Clays and Other Fillers in Preparation of Epoxy-Vinyl Ether Composites Using Radical-Induced Cationic Frontal Polymerization

Brecklyn R. Groce,[†] *Emma E. Lane,*[†] *Daniel P. Gary,*[†] *Douglas T. Ngo,*[†] *Dylan T. Ngo,*[†] *Fahima Shaon,*[†] *Jorge A. Belgodere,*[‡] *John A. Pojman*[†] *

[†]Department of Chemistry and the Macromolecular Studies Group, Louisiana State University, Baton Rouge, Louisiana 70803, United States

[‡]Department of Biological and Agricultural Engineering, Louisiana State University, Baton Rouge, Louisiana 70803, United States

*Correspondence to: John A. Pojman at john@pojman.com

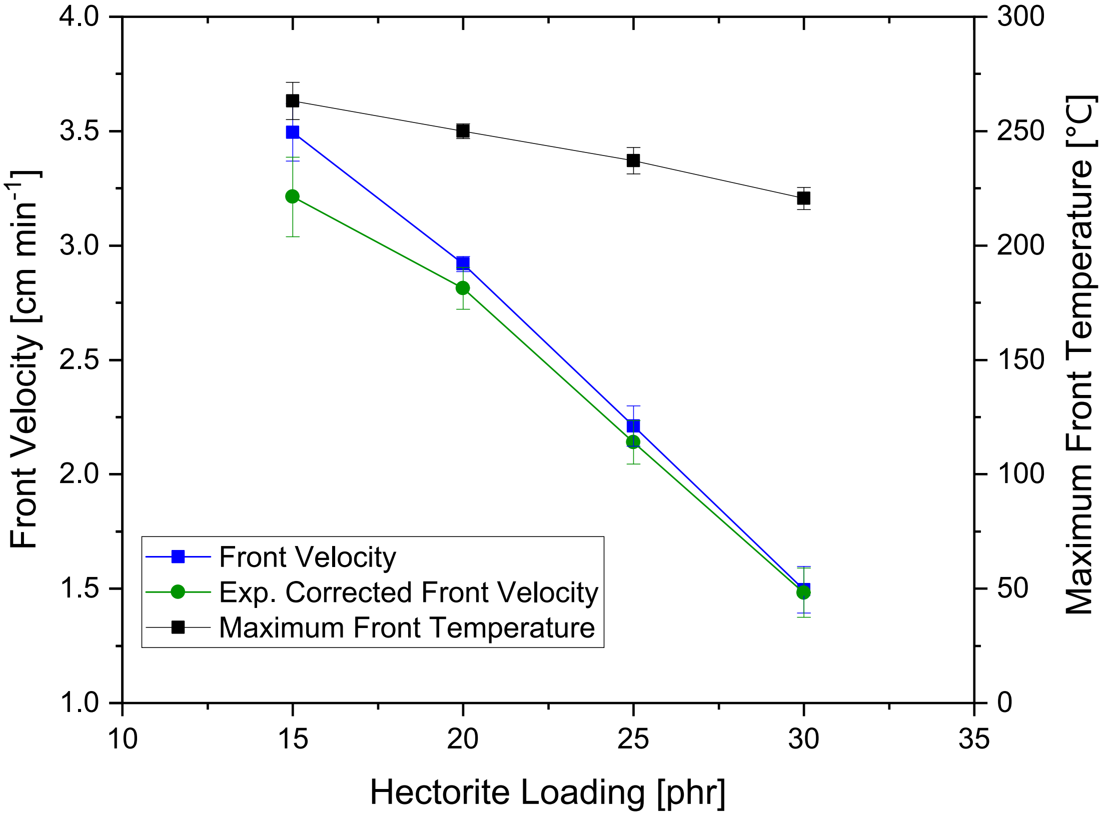

Figure S1: Front velocity, expansion corrected front velocity and maximum front temperature versus hectorite loading for a sample with 5 phr fumed silica added to a resin system of 25 wt% TEGDVE, 75 wt% TMPTE, 1 phr IOC-8 and 1 phr Luperox® 231. “Exp. Corrected Front Velocity” is velocity corrected for sample expansion during front propagation.

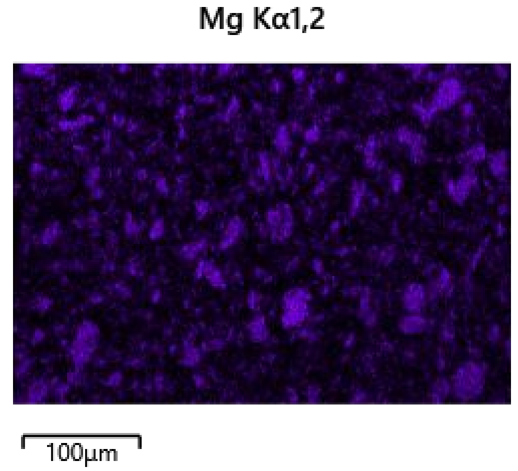

Figure S2: EDS map of Mg present in 60 phr talc composite.

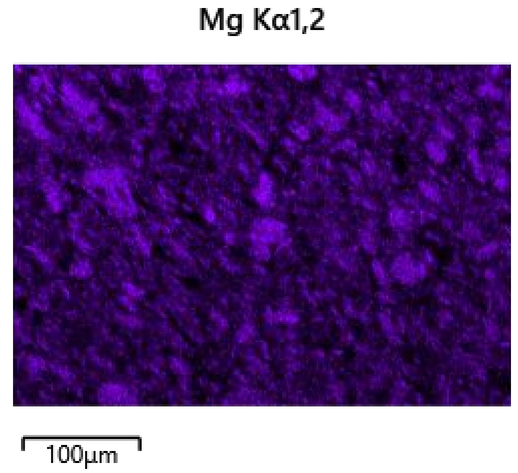

Figure S3: EDS map of Mg present in 120 phr talc composite.

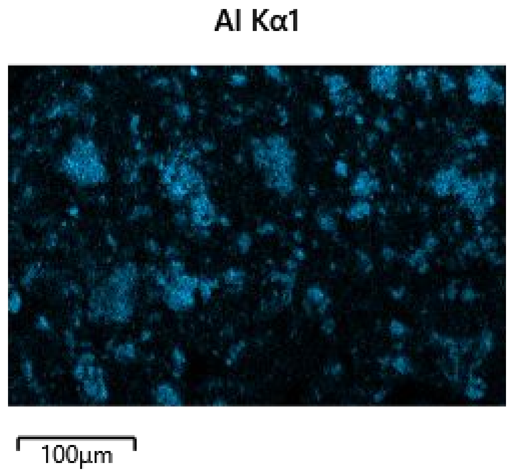

Figure S4: EDS map of Al present in 30 phr Ca-bentonite composite.

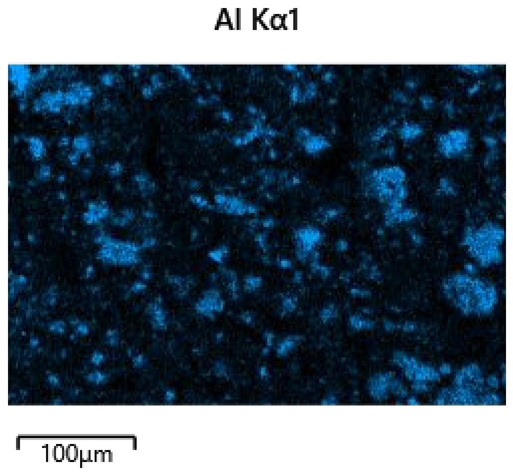

Figure S5: EDS map of Al present in 30 phr hectorite composite.

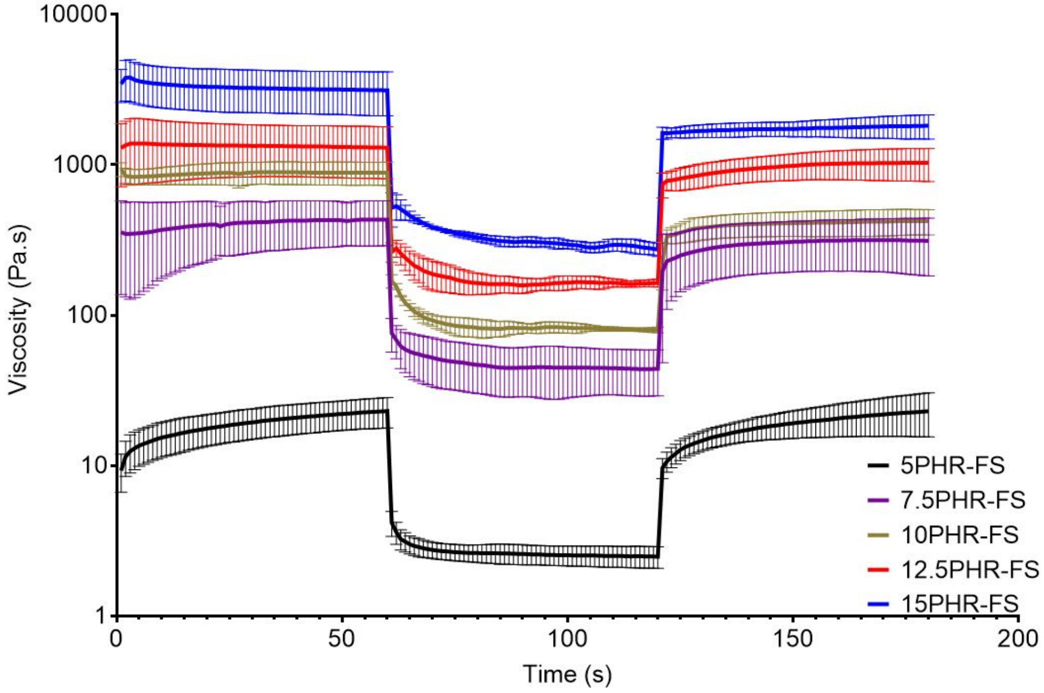

Figure S6: Viscosity profile of formulations containing the resin system of 25 wt% TEGDVE, 75 wt% TMPTE, 1 phr IOC-8 and 1 phr Luperox® 231 with increasing fumed silica loading. The first shear rate was 1 Hz, followed by 10 Hz and 1 Hz, with each shear rate held for 60 s at 25 °C.

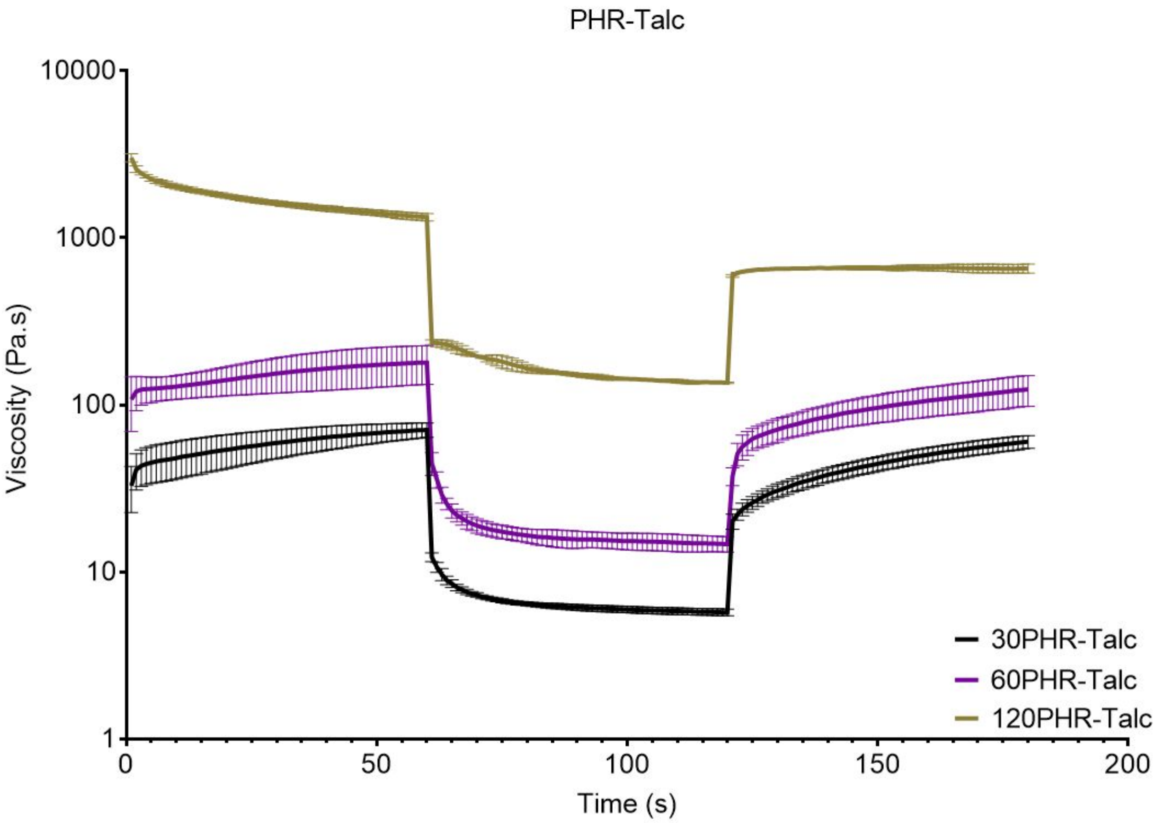

Figure S7: Viscosity profile of formulations containing the resin system of 25 wt% TEGDVE, 75 wt% TMPTE, 1 phr IOC-8 and 1 phr Luperox® 231 with 5 phr fumed silica and increasing talc loading. The first shear rate was 1 Hz, followed by 10 Hz and 1 Hz, with each shear rate held for 60 s at 25 ℃.

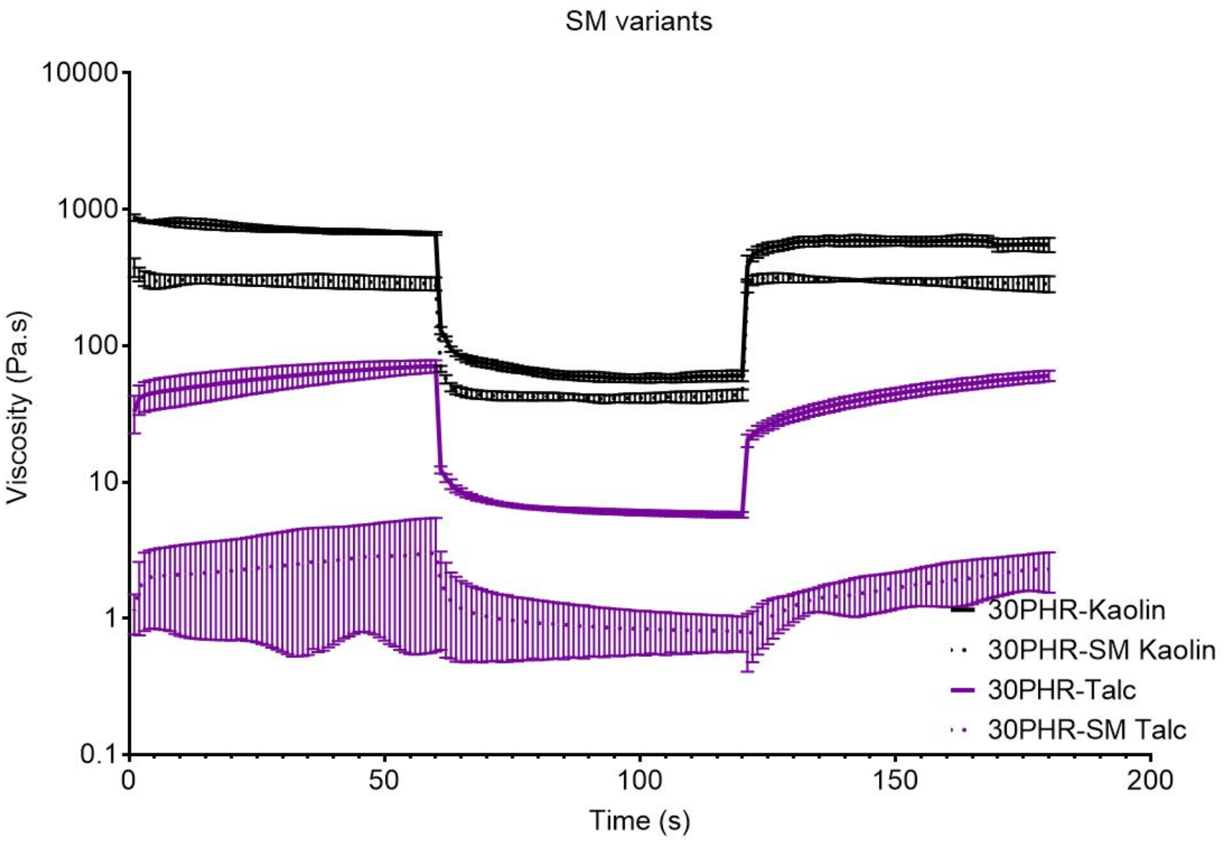

Figure S8: Comparisons of the viscosity profile of formulations containing the resin system of 25 wt% TEGDVE, 75 wt% TMPTE, 1 phr IOC-8 and 1 phr Luperox® 231 with 5 phr fumed silica and either 30 phr kaolin or talc, speed mixed (SM) versus hand mixed. The first shear rate was 1 Hz, followed by 10 Hz and 1 Hz, with each shear rate held for 60 s at 25 °C.

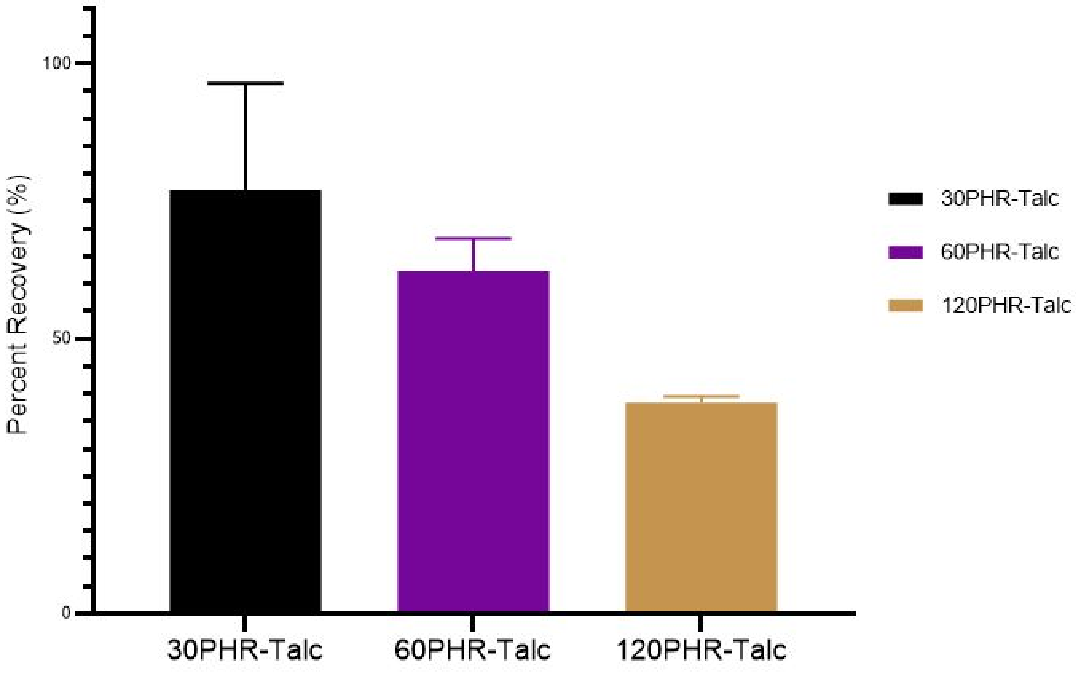

Figure S9: Percent recovery of viscosity after high shear of formulations containing the resin system of 25 wt% TEGDVE, 75 wt% TMPTE, 1 phr IOC-8 and 1 phr Luperox® 231 with 5 phr fumed silica and increasing talc loading.

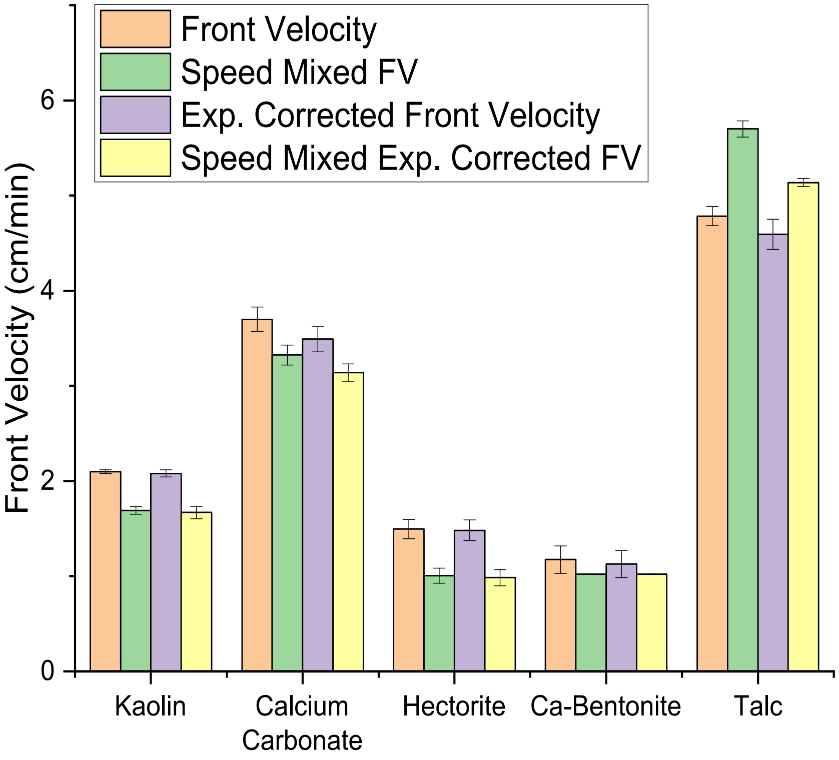

Figure S10: Front velocity and expansion corrected front velocity of hand-mixed and speed mixed samples with 5 phr fumed silica and 30 phr of the indicated filler added to a resin system of 25 wt% TEGDVE, 75 wt% TMPTE, 1 phr IOC-8 and 1 phr Luperox® 231. "Exp. Corrected Front Velocity" is velocity corrected for sample expansion during front propagation. Ca-bentonite speed mixed measurements are duplicates, not triplicates like other data points.

Detailed Procedure for Thermogravimetric Analysis of Drying of Fillers

A TA Instruments TGA 550 was used to analyze fillers thermogravimetrically. A Hi-Res™ Ramp 50 ˚C/min to 600 ˚C method, Resolution 4, Sensitivity 1 was used for each sample with standard aluminum pans under a nitrogen environment. This ramp method will alter the ramp rate dynamically depending on the observed weight change.

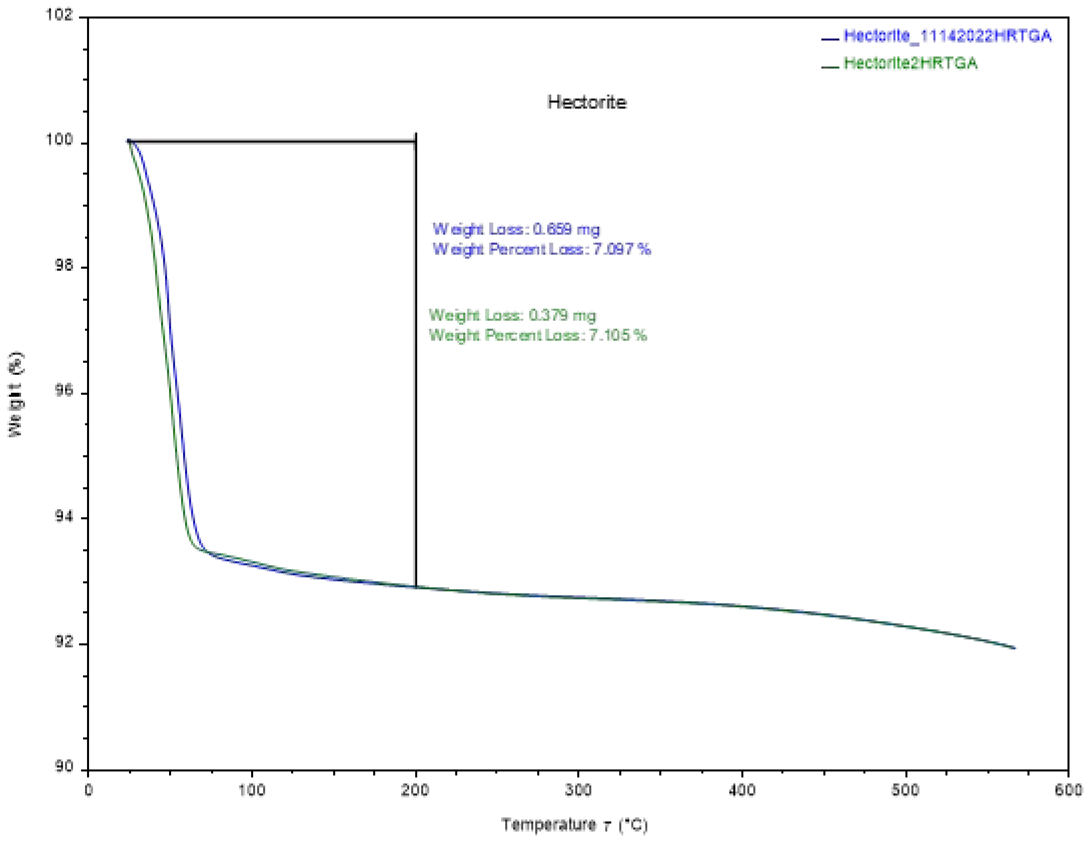

Figure S11. Thermogravimetric analysis (TGA) curve of Hectalite® 200 (hectorite) clay. Weight change calculated from starting temperature of approximately 25°C to 200°C.

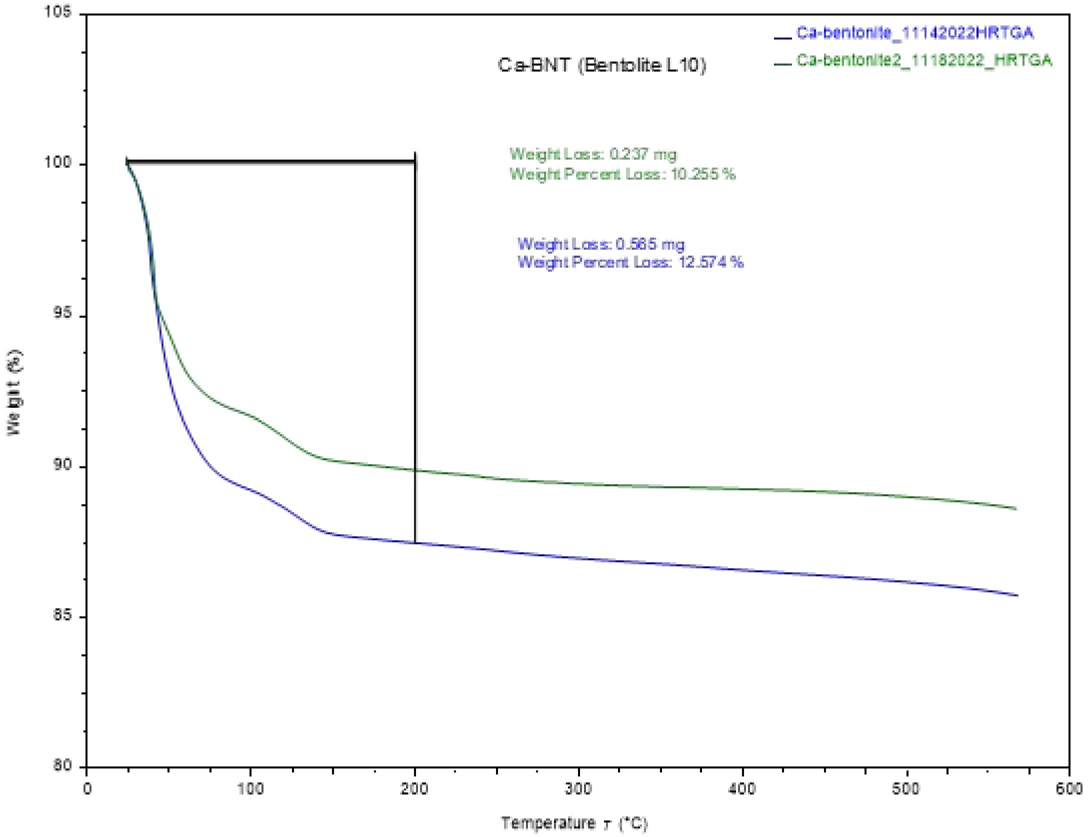

Figure S12. Thermogravimetric analysis (TGA) curve of Bentolite L10 (calcium bentonite) clay. Weight change calculated from starting temperature of approximately 25°C to 200°C.

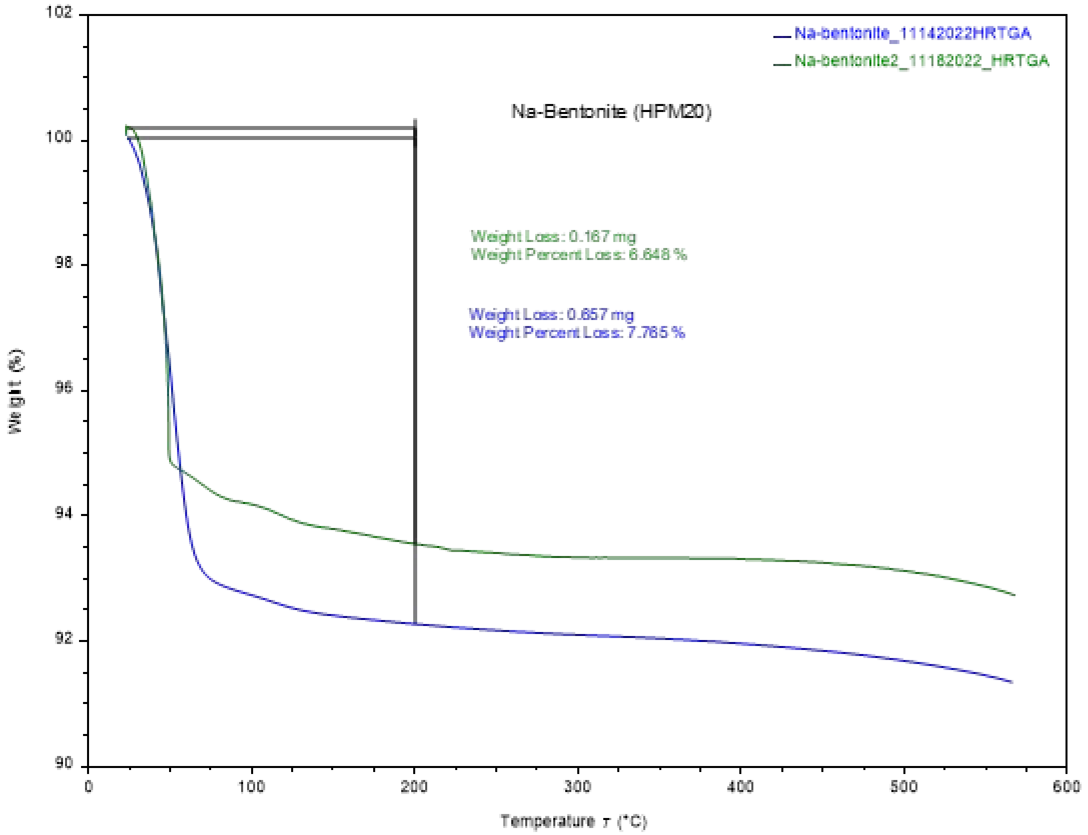

Figure S13. Thermogravimetric analysis (TGA) curve of bentonite HPM-20 (sodium bentonite) clay. Weight change calculated from starting temperature of approximately 25℃ to 200℃.

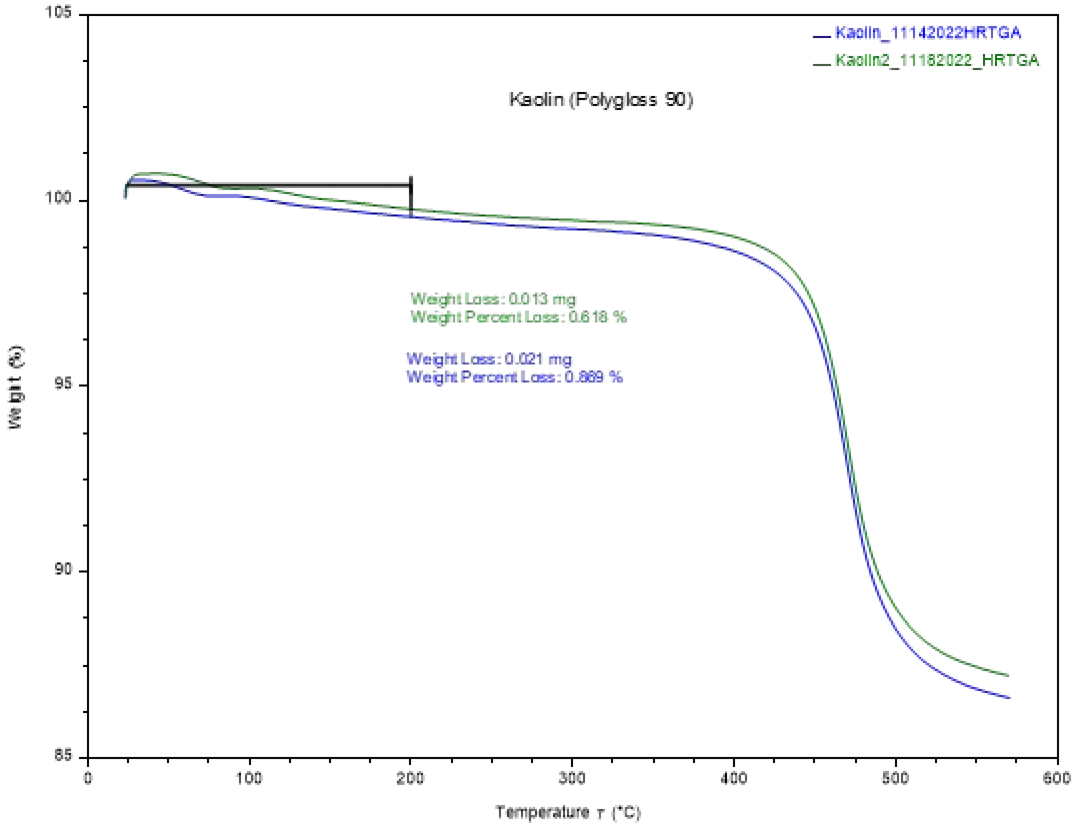

Figure S14. Thermogravimetric analysis (TGA) curve of Polygloss® 90 (kaolin) clay. Weight change calculated from starting temperature of approximately 25°C to 200°C.

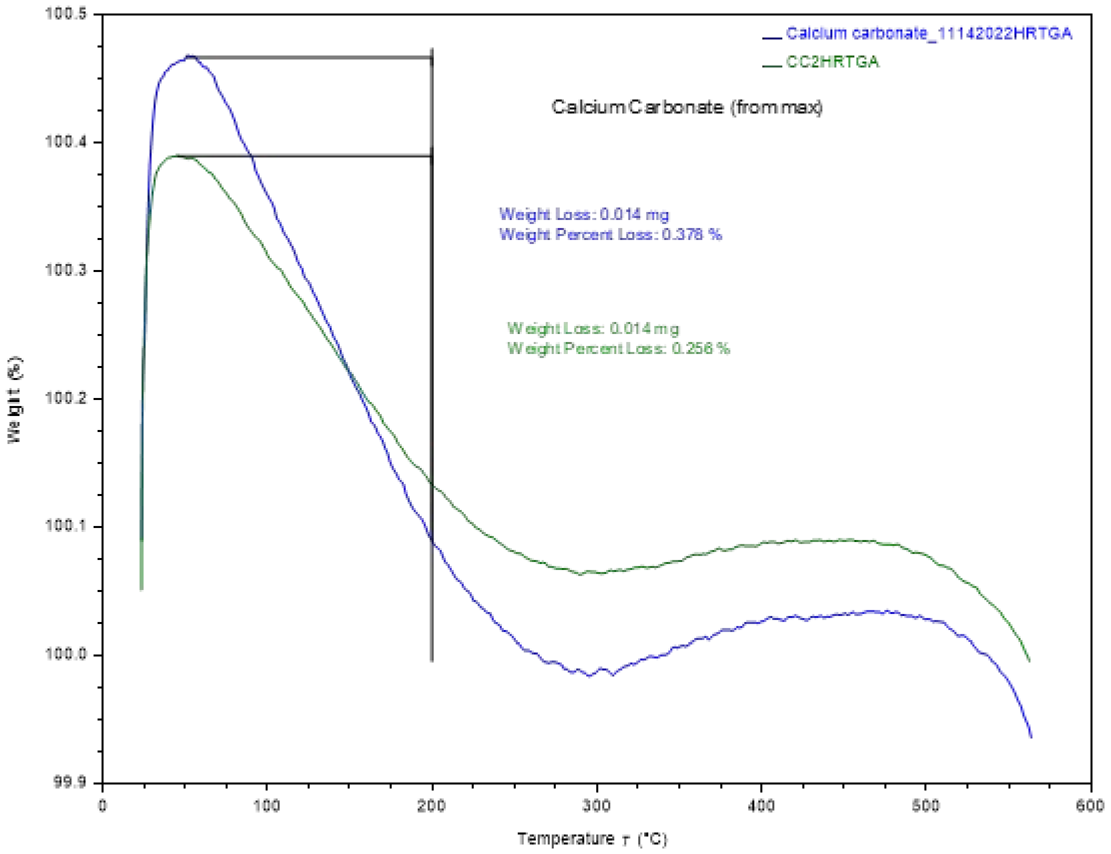

Figure S15. Thermogravimetric analysis (TGA) curve of Hubercarb® Q3 (calcium carbonate). Weight change calculated from starting temperature of approximately 50°C to 200°C.

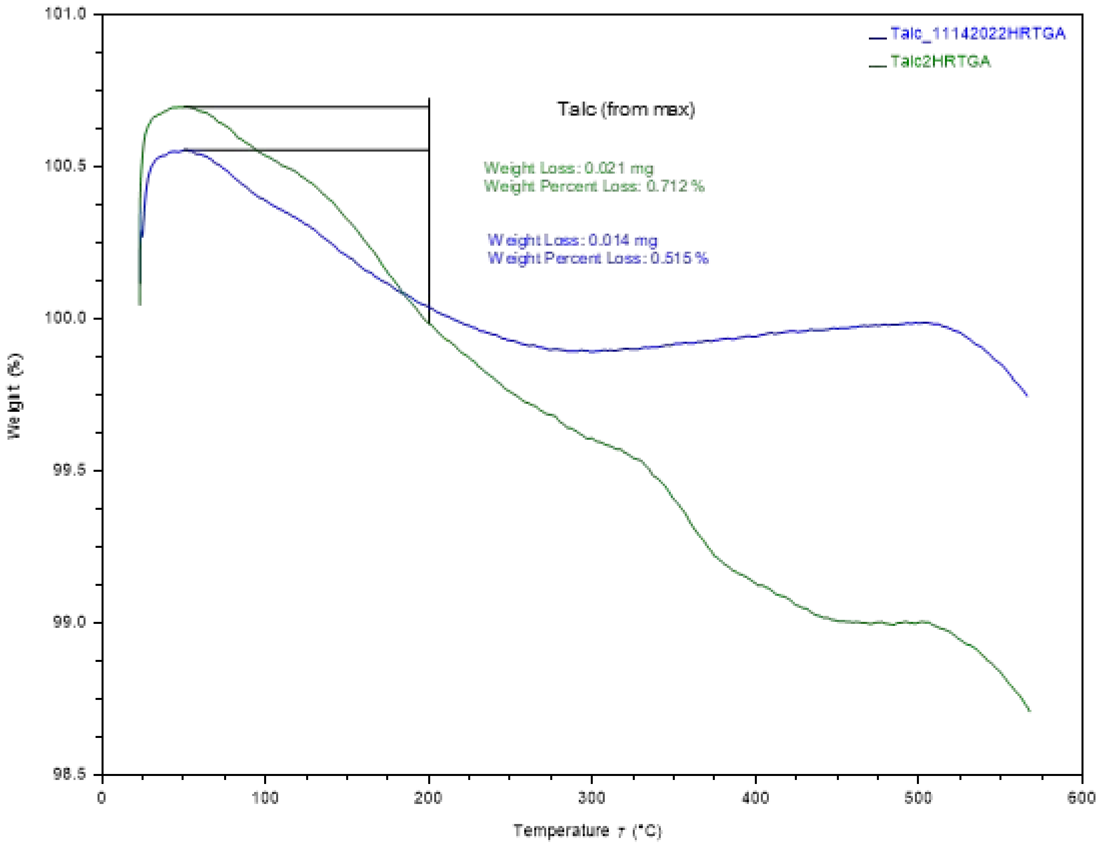

Figure S16. Thermogravimetric analysis (TGA) curve of talc. Weight change calculated from starting temperature of approximately 50°C to 200°C.

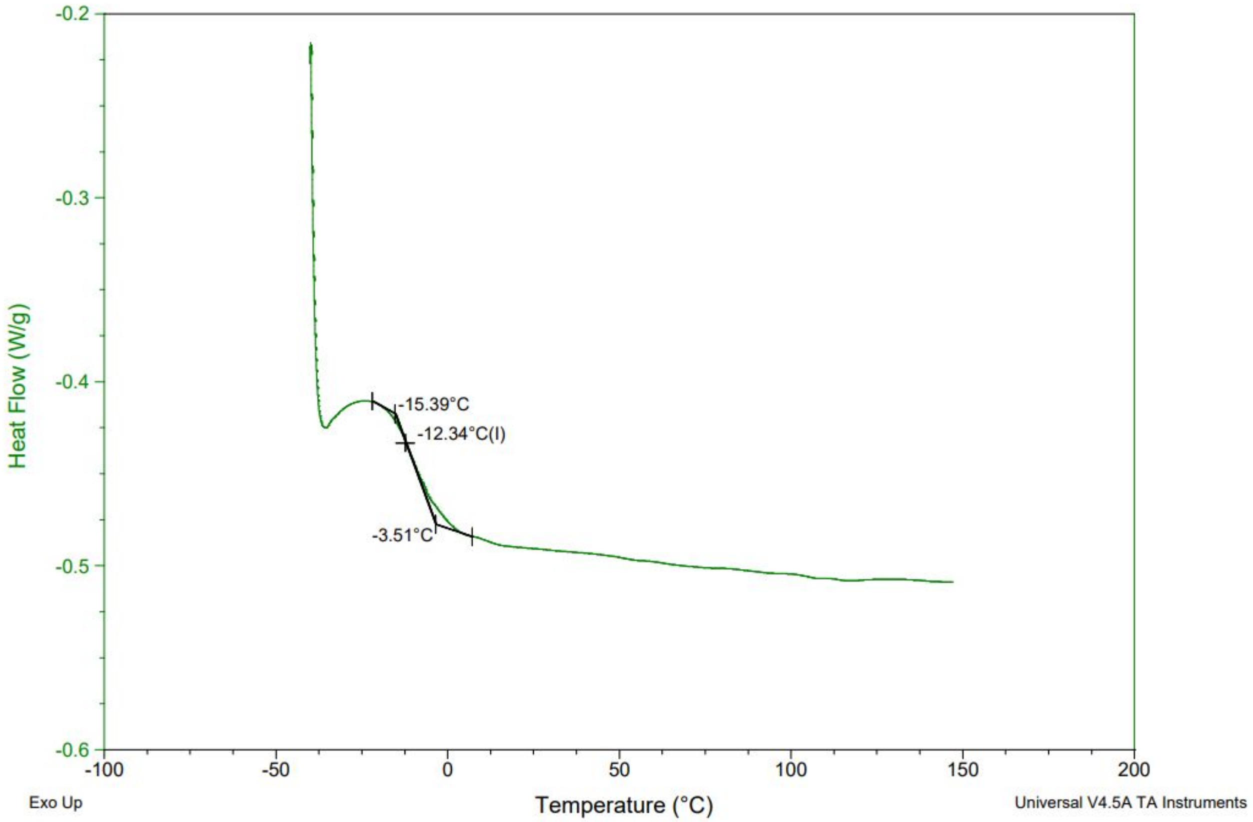

Figure S17. Example of the glass transition temperature calculation using TA Universal Analysis software for a composite sample containing calcium carbonate.

Table S1. Aspect ratio, surface area and sizing chemistry of filamentous fillers

	Aspect Ratio (*l*:*d*)	Surface Area (m^2 g^{-1})	Sizing (Coating)

Nyad® G[a]	15:1	0.4	None
Nyad® 1250[a]	3:1	3.2	None
10 ES Wollastocoat®[a]	3:1	3.2	Surface-modified, chemistry NP
Fibertec 520S[a]	20:1	2	Aminosilane
Fibertec Microglass 7204[a]	17:1	Not provided	Cationic
Fibertec Microglass 9114[a]	14:1	Not provided	Aminosilane
Fibertec FRM[a]	25:1	Not provided	None

[a]Provided by manufacturer, some data not provided

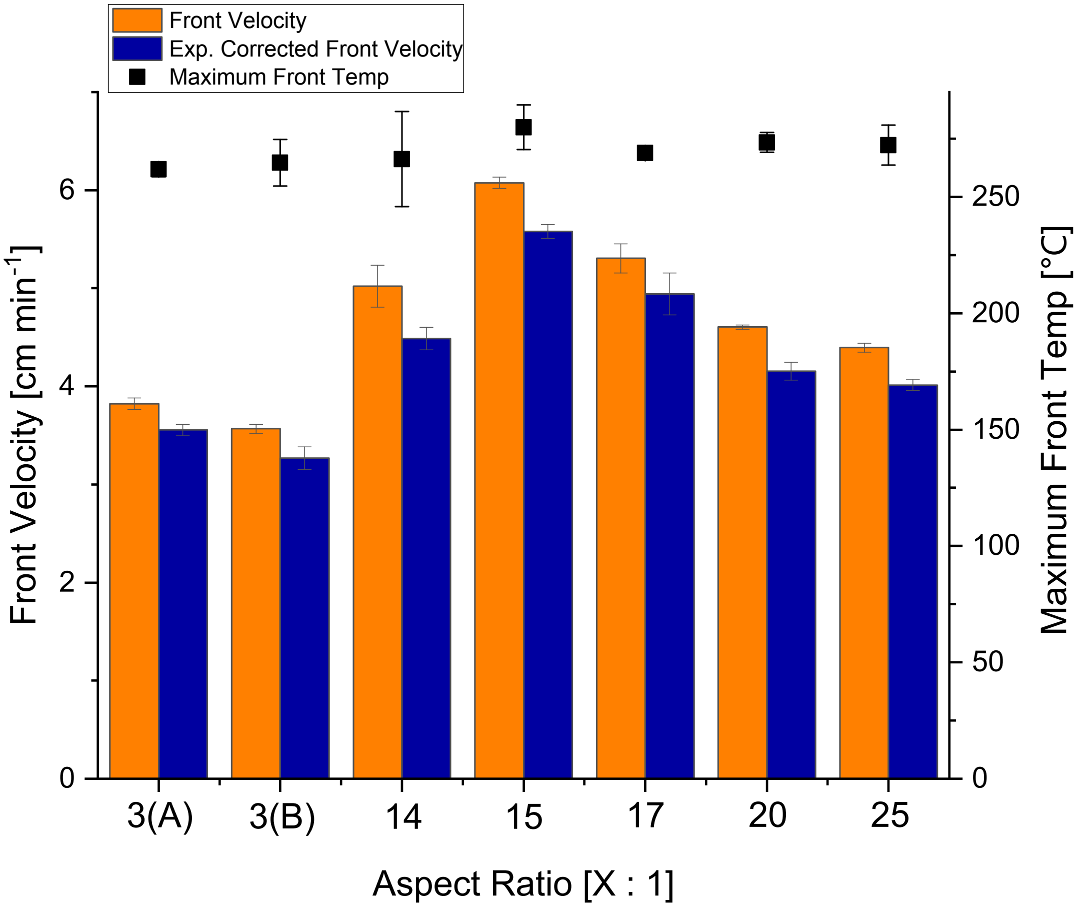

Figure S18. Front velocity and temperature as a function of filamentous filler aspect ratios. Resins contained 25 wt% tri(ethylene glycol) divinyl ether and 75 wt% trimethylolpropane triglycidyl ether, with 1 phr (parts per hundred resin) IOC-8 and Luperox® 231. 30 phr of each filamentous filler and 5 phr fumed silica was added (A = Nyad® 1250; B = 10 ES Wollastocoat®). "Exp. Corrected Front Velocity" is velocity corrected for sample expansion during front propagation.

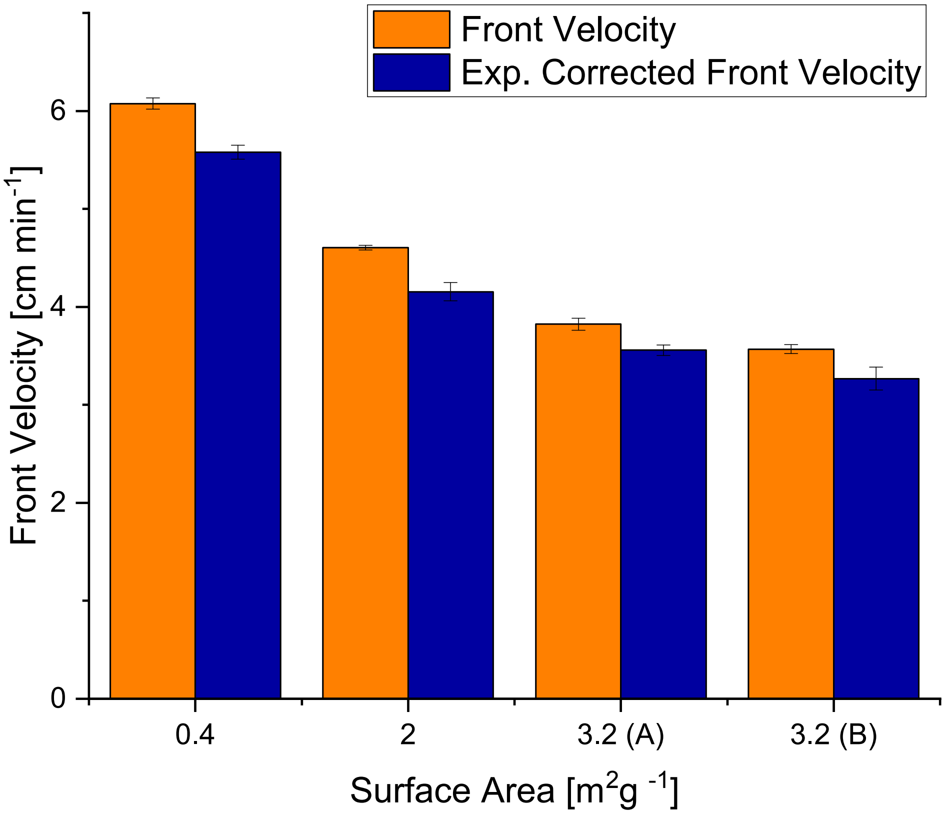

Figure S19. Front velocity and temperature as a function of filamentous filler surface area. Resins contained 25 wt% tri(ethylene glycol) divinyl ether and 75 wt% trimethylolpropane triglycidyl ether, with 1 phr (parts per hundred resin) IOC-8 and Luperox® 231. 30 phr of each filamentous filler and 5 phr fumed silica was added (A = Nyad® 1250; B = 10 ES Wollastocoat®). "Exp. Corrected Front Velocity" is velocity corrected for sample expansion during front propagation.

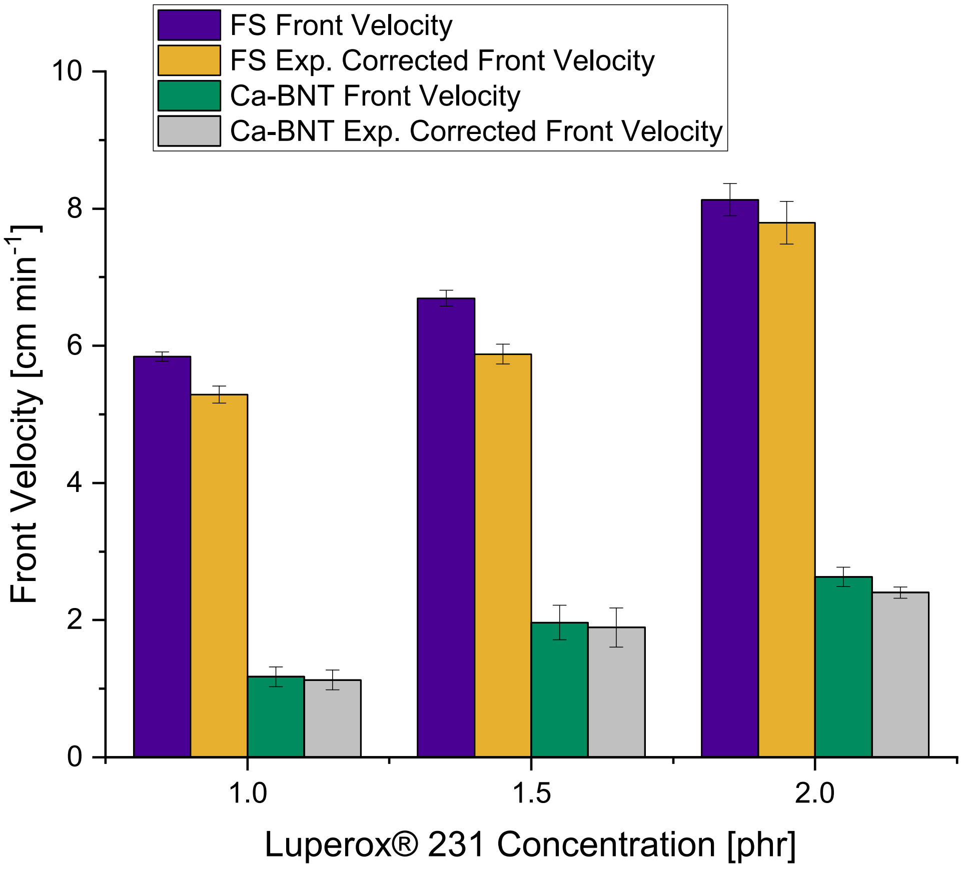

Figure S20. Effect of increasing Luperox® 231 concentration on front velocity for formulations containing 25 wt% TEGDVE, 75 wt% TMPTE and 1 phr IOC-8. Samples contained 10 phr fumed silica (FS) or 5 phr fumed silica with 30 phr Ca-bentonite. "Exp. Corrected Front Velocity" is velocity corrected for sample expansion during front propagation and "Ca-BNT" is Ca-bentonite.

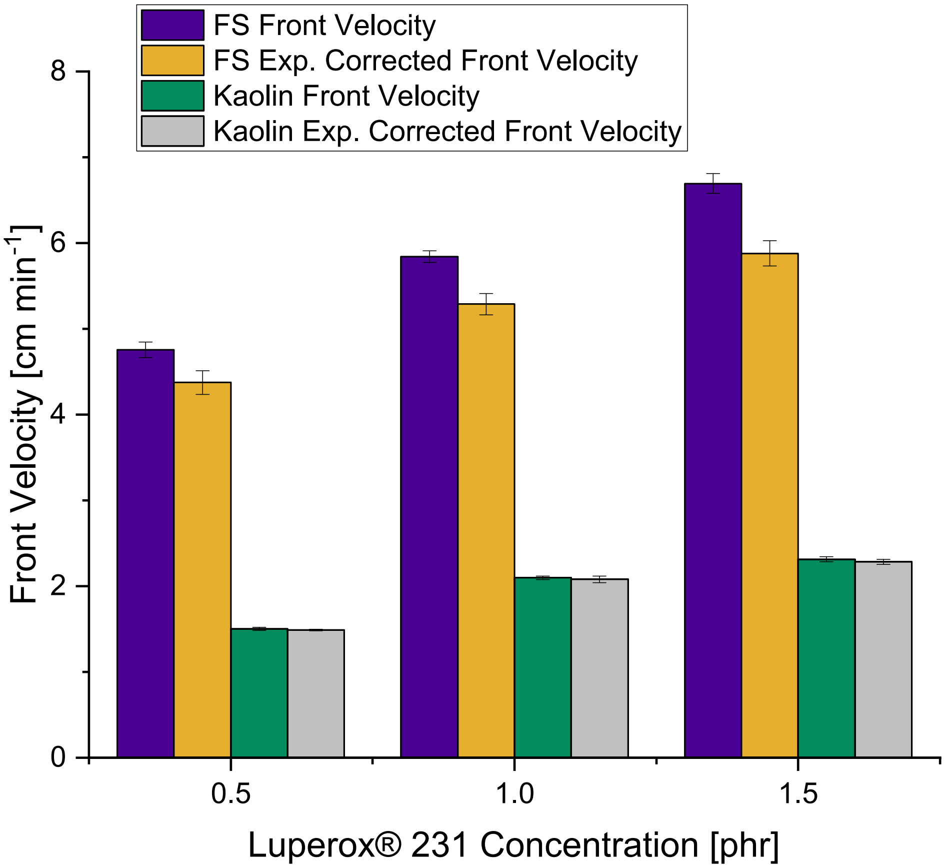

Figure S21. Effect of increasing Luperox® 231 concentration on front velocity for formulations containing 25 wt% TEGDVE, 75 wt% TMPTE and 1 phr IOC-8. Samples contained 10 phr fumed silica (FS) or 5 phr fumed silica with 30 phr kaolin. "Exp. Corrected Front Velocity" is velocity corrected for sample expansion during front propagation.

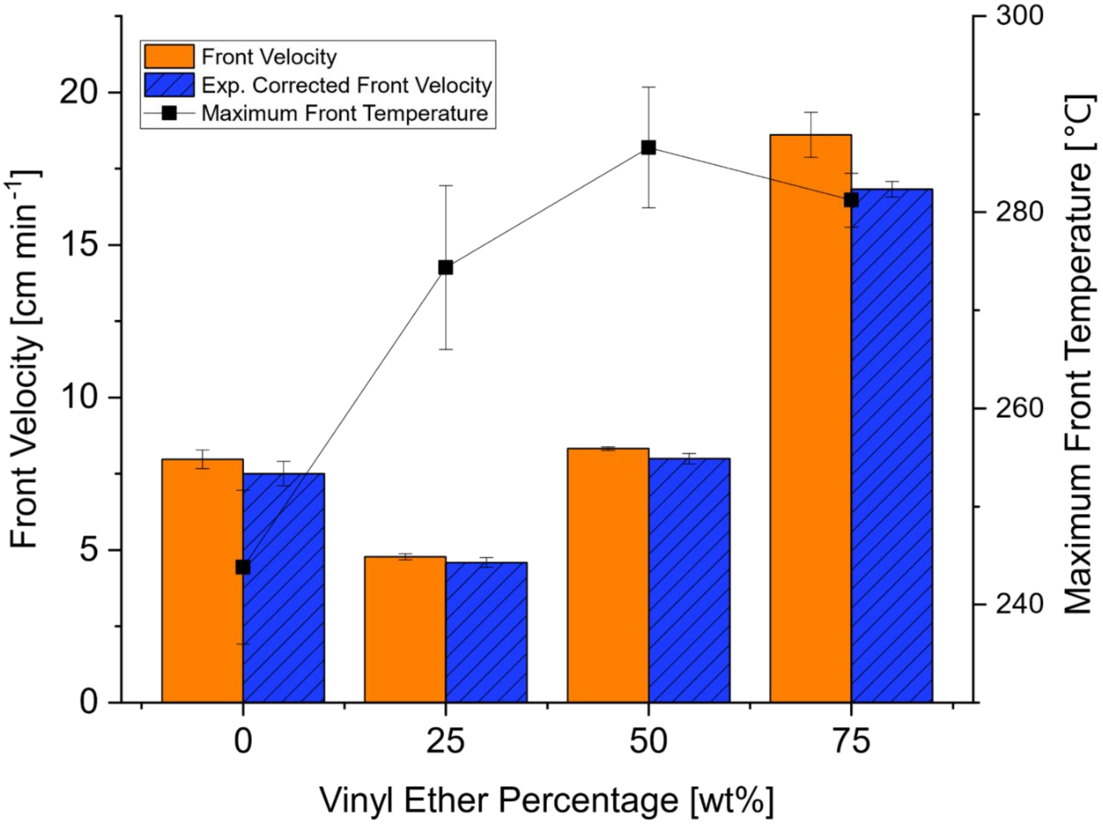

Figure S22: Front velocity and temperature as a function of TEGDVE percentage. Resins contained specified wt% TEGDVE and remainder wt% TMPTE, with 1 phr (parts per hundred resin) IOC-8 and Luperox® 231, and 30 phr talc with 5 phr fumed silica. "Exp. Corrected Front Velocity" is velocity corrected for sample expansion during front propagation.

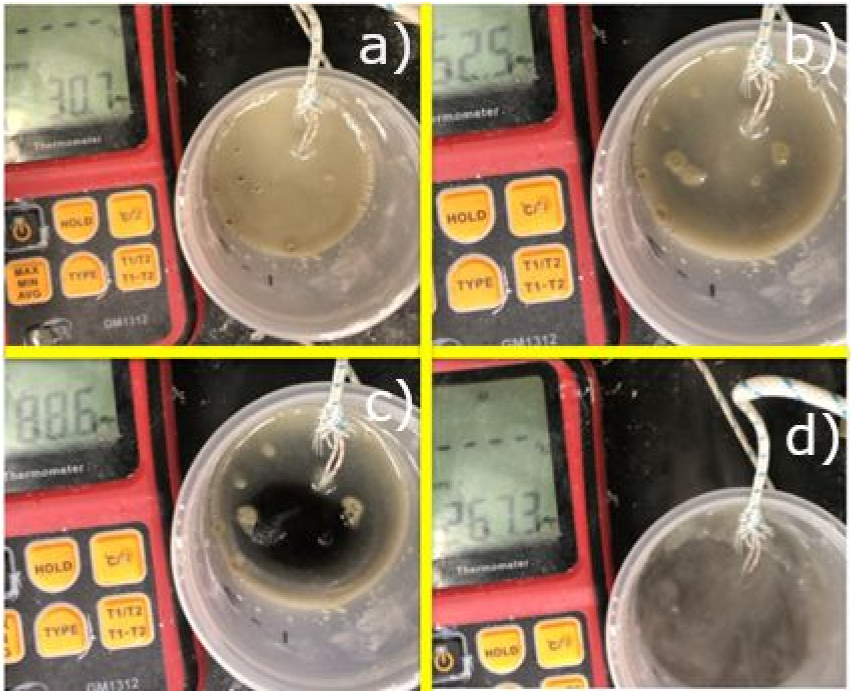

Figure S23: Spontaneous polymerization over time of a 25 wt% TEGDVE, 75 wt% TMPTE and 1 phr IOC-8 with 1 phr Luperox® 231 formulation via addition of MMT K10. The time intervals shown are: a) 80 seconds; b) 335 seconds; c) 435 seconds; d) 465 seconds.
